# PROTOCOL: The Effects of Land Management Policies on the Environment and People in Low‐ and Middle‐Income Countries: A Systematic Review

**DOI:** 10.1002/cl2.70062

**Published:** 2025-10-27

**Authors:** Pierre Marion, Ingunn Storhaug, Sanghwa Lee, Claudia Romero, Constanza Gonzalez Parrao, Birte Snilstveit

**Affiliations:** ^1^ International Initiative for Impact Evaluation (3ie) London UK; ^2^ Economics Department, Business School University of Sussex Brighton UK; ^3^ Tropical Forests and People Research Centre University of the Sunshine Coast Sunshine Coast Australia

## Abstract

Addressing the climate change and biodiversity loss crises while ensuring livelihoods are not negatively affected is a matter that requires urgent action. A recently published Evidence Gap Map (EGM) identified no recent systematic reviews on land management interventions. Drawing from this EGM, the review aims to examine and synthesise the latest evidence on what works, how, and at what cost to improve environmental and human welfare outcomes in land management in low‐ and middle‐income countries. We will address the following research questions: (1) What are the effects of protected areas, land rights and decentralisation interventions on environmental and poverty outcomes? Do effects vary by population, location, or other factors? (2) What are the barriers and enablers that impact the effectiveness of these interventions? (3) What is the cost‐effectiveness of these interventions? The set of interventions will be based on the studies identified in the EGM, and we will search, appraise and synthesise additional evidence on influencing factors and cost data.

## 1 Background

### 1.1 The Problem, Condition or Issue

The World Economic Forum estimated that more than half of global economic activity (USD 44 trillion) relies heavily on natural resources (World Economic Forum WEF 2020). Local communities and individuals' livelihoods in low‐ or middle‐income countries (LMICs), especially the most vulnerable populations, often directly depend on exploiting land for livestock, hunting and farming (Organisation for Economic Co‐operation and Development [OECD] 2023; Intergovernmental Panel on Climate Change [IPCC] 2023; Cannizzo et al. 2024), revealing the existence of trade‐offs between the maintenance of natural systems with the achievement of human wellbeing aspirations. It is estimated that 20% of the world's population relies on wildlife for income and food (Intergovernmental Science‐Policy Platform on Biodiversity and Ecosystem Services [IPBES] 2019). Access to traditional medicines and nutritional resources is also closely linked to land and forest resources and ecosystem services (IPCC 2023; OECD 2023).

Land resources are currently depleting at an alarming rate globally. The loss of land's fresh water, soil richness and ecosystems has devastating impacts on the climate, biodiversity and human wellbeing. It is estimated that almost 75% of global land has been altered, and at least 20% of global land has been degraded, according to the United Nations Convention to Combat Desertification (UNCCD 2022). Biodiversity outcomes have also significantly deteriorated in the last few decades. An estimated one million species are at risk of extinction (IPCC 2023; Dasgupta 2021; Secretariat of the Convention on Biological Diversity 2020). According to IPBES (2019), animal pollination continues to decline, the population of vertebrate species has declined by 60% since 1970, and the world's forest area has diminished by 32%.

In most natural systems in LMICs, climate change furthers the depletion of land resources. Global temperatures have risen since the start of the Industrial Revolution and are likely to exceed the goals of the Paris Agreement by 2100 (IPCC 2023). LMICs are facing more severe and frequent climatic shocks and natural disasters, such as floods, droughts, heat waves, and wildfires, than in other countries (World Meteorological Organization [WMO] 2021). These shocks also progressively reduce households' income‐generating capacities, impacting livelihoods and welfare, possibly even leading to poverty‐traps (Carter and Barrett 2006; Eichsteller et al. 2022) and further exposure to climate change and other risks (e.g., Barbier 2025). Uncontrolled rising temperatures also affect the ecosystems and biodiversity of the local environment by reducing the ability of species to adapt (Dasgupta 2021; IPBES 2019) and potentially leading to other changes, such as the emergence of conditions that favour the spread of non‐native invasive species (Weiskopf et al. 2020). With rising temperatures, animals and plants migrate to higher elevations or latitudes, often moving towards the poles, which also affects ecosystems (United Nations [UN] 2024). These changes exacerbate the intense pressure of ever‐increasing human populations that results in land use change and resource degradation.

The use and management of land is central to addressing the climate and biodiversity crises. Deforestation releases carbon into the atmosphere and reduces the ability of ecosystems to sequester carbon through persisting degradation (Dasgupta 2021; IPBES 2019). Moreover, in many LMICs, land is often not formally demarcated or registered, which can facilitate land conflict or dispossession to control land resources (UNCCD 2022). Land insecurity can impose costs on communities and individuals to protect land use and encourage short‐term value‐extracting activities (Besley 1995) and even land‐grabbing by powerful actors, including the state, with uses that lead to resource degradation (Carrero et al. 2022).

Without direct intervention, land management and climate change will interconnectedly worsen. These changes can seriously threaten livelihoods and wellbeing (Armand and Kim Taveras 2022). Effective, fair and inclusive land management interventions are urgently needed. Improved land use can slow down the negative cycle of depleting land resources and climate change by better preserving, conserving and restoring water, soil and biodiversity environments. These efforts, in turn, can contribute to climate change mitigation and adaptation and, therefore, support livelihoods and wellbeing.

### 1.2 The Intervention

In this review, we will synthesise and critically appraise the evidence on the effects of three types of land management interventions in LMICs: (1) community‐based or decentralisation of land management and monitoring, (2) land rights and (3) protected areas. This review relies on the Climate Change and Biodiversity Evidence Gap Map (CCB EGM; Marion et al. 2024), drawing from its intervention categories and included studies.

a. Community‐based or decentralisation of land management and monitoring
  This intervention involves transferring the management and decision‐making authority over natural resources from national to regional and/or local actors. Community participation may take the form of fully or partially decentralised land management, monitoring of external managerial authorities, or reporting on natural resource use. This intervention type aims to exploit local knowledge and presence to preserve and sustainably use natural resources. Examples include community‐based forest management, community representation in forest managerial organs (e.g., community councils), and crowdsourced reporting and monitoring systems (e.g., wildfire risk management, timber tracing).
b. Land rights
  We define land rights as a formal registration of land, either through the conversion of communal or non‐demarcated rural land to freehold title, or the statutory recognition of customary or communal rural land rights. Land rights are registered and recognised by a central authority, enabling right holders to legally defend and enforce their access and control of this land. In turn, right holders can fully determine land use in terms of activities (e.g., pasture, forestry, agriculture, left fallow, leasing, sale of land), investment level, and hiring of labour.
c. Protected areas
  The International Union for Conservation of Nature (IUCN) describes a protected area as ‘a clearly defined geographical space, recognised, dedicated and managed, through legal or other effective means, to achieve the long‐term conservation of nature with associated ecosystem services and cultural values’ (Dudley 2008, 8). Protected areas have defined boundaries, formally recognised under statutory civil law or traditional rules aiming to influence natural systems and human behaviour to protect, maintain, and restore ecosystems and biodiversity. Several categories of protected areas exist, ranging from conservation activities (such as wildlife conservation) to a more comprehensive area management for the sustainable use of resources. Protected areas are often essential to most national biodiversity conservation strategies in LMICs (Jonas et al. 2024; Lopoukhine and de Souza Dias 2012).

### 1.3 How the Intervention Might Work

This review is based on rich theoretical and empirical literature describing how these three land management interventions may affect both the environment and people in positive or negative ways. Importantly, interventions' implementation could also generate situations where positive changes in one dimension (i.e., environmental) have demonstrably burdened the other (i.e., human wellbeing, or vice versa), leading to trade‐offs. More often than not, these trade‐offs have differing consequences in members of the same communities as a function of existing or exacerbated inequalities and vulnerabilities related to gender, age, and other aspects.

**Figure 1:**
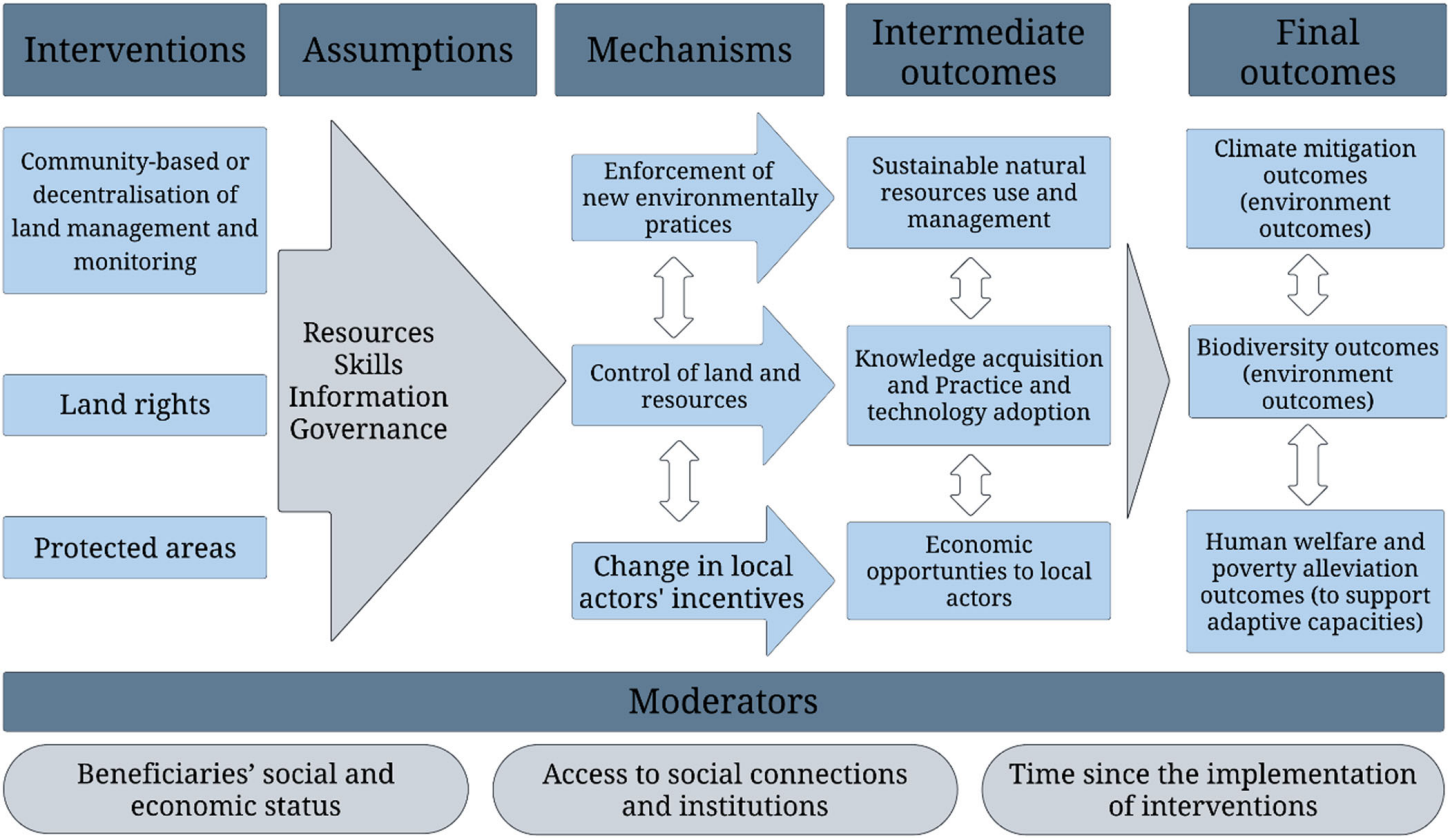
Conceptual framework for the review of land management interventions. Moderators include mechanisms through which elements of the interventions act (i.e., drivers). Vertical arrows are indicative of some but not all possible interactions between mechanisms and between intermediate and final outcomes.

What needs to be established and synthesised through this review are the conditions under which these outcomes along the change process have been achieved and the nature of contextual and other factors that served as enablers or impediments of the desired change (i.e., assumptions). In this section, we outline the conceptual framework used in this review, discussing the key expected outcomes, mechanisms of change, assumptions, and possible unintended consequences of these three interventions (e.g., negative spillovers or leakage both through time and space). Figure 1 provides a graphical representation of this framework across these three land management interventions.

#### 1.3.1 A Conceptual Model for Desired Long‐Term Change

This review aims to explore how these interventions rely on mechanisms that could lead to impacts associated with improved climate change mitigation and adaptation conditions, favourable biodiversity status, and enhanced human well‐being conditions. Intermediate outcomes to achieve longer‐term desired changes relate, but are not exclusive to, increased skills of decision‐makers and resource managers, including local communities. Likewise, plausible resulting intermediate outcomes refer to improved resource management and better economic prospects for local actors, such as encouraging the dissemination of knowledge and the adoption of environmentally friendly and innovative practices and technologies. These three interventions can also promote sustainable use and management of natural resources and offer new economic opportunities.

In turn, biodiversity outcomes may improve. These include the preservation and development of habitat integrity and species diversity (Brodie et al. 2023; Justin Nowakowski et al. 2023) and maintenance and enhancement of ecosystem services (Samii et al. 2015; Lawry et al. 2012), sometimes leading to direct and indirect health benefits (MacKinnon et al. 2019). Furthermore, additional evidence has also suggested that the sustainable use of resources and improved biodiversity outcomes can contribute to greater adaptation to and mitigation of the adverse effects of climate change and biodiversity loss (Cannizzo et al. 2024; Duncanson et al. 2023).

Previous research has also linked land management interventions with poverty alleviation in the context of these two inter‐connected crises (Turner et al. 2012; Samii et al. 2015). In these cases, their implementation has helped reduce social tensions and improve overall well‐being (Miller et al. 2021, 2022; Hajjar et al. 2021). Therefore, changes in one of these outcomes can have implications for other outcomes. However, the magnitude of these changes is likely to vary by the characteristics of those affected (i.e., positively or negatively) by interventions' implementation. Beyond issues related to actors' social and economic status, including power and other asymmetries inherent to each group based on gender, age, ethnicity and other factors, access to social connections, institutions, and resources, as well as the time elapsed since the implementation of the interventions, will shape their outcomes and ultimate impacts.

#### 1.3.2 Mechanisms

These three land management interventions can promote sustainable use and management of natural resources through several mechanisms. First, these interventions can improve the enforcement of sustainable land practices. Protected areas impose specific punishment penalties against illegal deforestation, hunting or over‐exploitation (Koch et al. 2019), which in turn can facilitate the preservation and restoration of the local environment (Pullin et al. 2013; Dudley 2008; Schmidt–Soltau 2003; West et al. 2006). Similarly, with land rights and decentralised land management, enforcement is more likely than in scenarios of open access, and the ‘tragedy of the commons’^1^ is less likely to occur when land is individually owned (Rakotonarivo et al. 2023), or when incentives, even implicit, exist that will foster and in some cases maintain traditional collective resource management practices through the establishment of social institutions, rules and norms. In decentralised systems, resources are restricted to local users, bringing the consequences of land management and use closer.

Second, land rights and decentralisation of land management can contribute to sustainable use and management of natural resources by giving individuals and local communities control of resources. Local actors are often most knowledgeable about the local conditions and can best monitor different situations (Lawry et al. 2012, 2017; Samii et al. 2015). Compared to central authorities, they are most likely to adapt land management practices and technologies to local needs.

Control of local land and resources can also strengthen local actors' incentives to use natural resources sustainably. These three interventions can reduce land insecurity, thereby eliminating land conflict costs and encouraging long‐term land investments (Besley 1995). When there is control of land and resources, the sustainability of resources' use is aimed to maximise long‐term gains (Lawry et al. 2012; Gibson et al. 2000; Barbier and Burgess 2001; Bohn and Deacon 2000). Decentralisation of land management may change local groups' mutual interactions and result in the design of social norms and institutions that could implement informal sanctions or social pressures, aligning individual actions with group interests (Agrawal and Ostrom 2001; Baragwanath et al. 2023; Agrawal et al. 2023). In other cases, elite capture and power‐grabbing may result (Persha and Andersson 2014).

These three interventions can also provide economic opportunities and potential welfare gains for local actors, as their livelihoods often rely on land and natural resources (OECD 2023; IPCC 2023; Cannizzo et al. 2024). These opportunities can, for example, translate into ecotourism in and around protected areas and other land utilisation and development from land rights and decentralised land management. Protected areas are often accompanied by infrastructure and road developments that facilitate economic activities within local communities (Ferraro and Hanauer 2011; Zhang et al. 2023). Land rights can be used as collateral and improve access to credit (Besley 1995; Fenske 2011), with which landowners can invest in sustainable land production and use. Land ownership can also provide greater control to individual households in decisions linked to selling, leasing out, sharecropping land or hiring labour (Lawry et al. 2017). As pointed out earlier, some of these opportunities are not equally accessible to all sectors of communities. Some local actors, given inherent vulnerabilities (e.g., age, disability) and internal political economies of power, may suffer costs.

#### 1.3.3 Assumptions and Unintended Consequences

The success of these three interventions relies on assumptions linked to resources, skills and information access, and adequate governance.^2^ For example, Lessmann et al. (2024) highlighted a substantial funding deficit hindering the effective management of protected areas. Corrupt, under‐resourced or ineffective control of protected areas and land can also prevent improvements in environmental and human outcomes (Blackman et al. 2017; Liu et al. 2001; Kahsay and Bulte 2021). Failures within credit, inputs, or outputs markets or information asymmetries across local actors can compromise the potential improvements of these interventions.

It is also important to acknowledge that these interventions may have unintended consequences for land and people. Protected areas, and to a lesser extent, decentralisation of land management, may reduce local economic welfare by restricting land use choices and constraining human actions as it limits local actors' ability to use land for agriculture, logging, and hunting (Schmidt–Soltau 2003; West et al. 2006; Pullin et al. 2013; Samii et al. 2015). Although protected areas may increase income and employment from ecotourism (Pullin et al. 2013; den Braber et al. 2018; Balmford et al. 2009), ecotourism may also displace other existing jobs or increase local prices (Robalino 2007). There have been cases of local communities being forced out so protected areas can be designated (Brockington et al. 2006; Robalino 2007; Lunstrum and Ybarra 2018).

These interventions may also exacerbate income inequalities if benefits are captured by large commercial enterprises or more powerful or wealthier households (Samii et al. 2015; Kahsay and Bulte 2021). The implementation of these interventions may also be co‐opted by interests seeking private short‐term benefits, both by internal actors (e.g., elite capture: Jagger et al. 2014; Persha and Andersson 2014) and by actors external to the communities (i.e., carbon cowboys: Rifai et al. 2015; West et al. 2023). If these interventions only benefit the targeted areas, inequality between protected and non‐protected areas or between areas with and without land rights may increase (Lawry et al. 2017; Kandel et al. 2022; den Braber et al. 2024). Because of the contested nature of lands, lack of regional planning and often inconsistency in governmental actions can lead to social‐environmental failures (Kennedy et al. 2023). Lastly, the coexistence of different interventions in the same location and time can also affect their intended objectives.

Likewise, these land interventions may not always encourage long‐term incentives to adopt environmentally friendly practices and to use resources sustainably. Local actors' incentives are likely to change over time based on external factors, such as the implementation of new development programmes or changes in regional and international markets. Trade‐offs between the preservation of the local environment and economic aspirations may emerge, for example, when decentralisation of land management or land rights encourages transferring negative externalities to neighbouring areas (i.e., negative spillovers or leakage). Community‐level land management groups may also be incentivised to increase pollution if they know they can transfer it downstream to other communities and there is no national or regional enforcement (Lipscomb and Mobarak 2017). Land rights holders' incentives may not always follow practices that are aligned with long‐term land and biodiversity conservation. For example, a steep increase in a crop price may encourage landowners to convert forests into agricultural land to make it more efficient and profitable (Wood and Walker 2000; Godoy et al. 2001). Another example of trade‐offs across these interventions is credit policies to increase access to funds to rural people, which may lead to an inadvertent increase in deforestation (Assunção et al. 2020).

### 1.4 Why It Is Important to Do This Review

Many governments and international organisations are promoting these land management interventions to mitigate and adapt to the effects of climate change and biodiversity loss. Target 3 of the Kunming‐Montreal Global Biodiversity Framework is to effectively manage and equitably govern, protect and conserve at least 30% of the Earth's land and water by 2030 (United Nations Environment Programme World Conservation Monitoring Centre [UNEP‐WCMC] and International Union for Conservation of Nature [IUCN] 2024). Protected areas are becoming increasingly prevalent in national conservation policies. Approximately 18% of the world's terrestrial and inland water areas are now covered by the more than 300,000 reported protected areas (UNEP‐WCMC and IUCN 2024). Similarly, governments frequently transfer land and forest management and decision‐making to local communities. In LMICs, 30% of forests are managed by communities (i.e., indigenous people and local communities) according to the Rights and Resources Initiative (2021).

National and international organisations are also incentivised to support these land management interventions as they are linked to the Sustainable Development Goals (SDGs), especially SDGs 13 and 15 (IUCN 2022). SDG 13 aims to ‘take urgent action to combat climate change and its impacts’ (UN Department of Economic and Social Affairs [UNDESA] 2020, 15), and SDG 15 is to ‘protect, restore and promote sustainable use of terrestrial ecosystems, sustainably manage forests, combat desertification, and halt and reverse land degradation and halt biodiversity loss’ (UNDESA 2020, 17).

With this increased interest, the number of studies evaluating the effects of these interventions has therefore grown at a fast pace. Following a systematic search and screening of studies, the CCB EGM (Marion et al. 2024) identifies the available impact evaluations on the effects of climate change and biodiversity interventions (including these three intervention types) on environmental and human wellbeing outcomes in LMICs. In this EGM, 75% of the evaluations assessing the effects of these three intervention types were published since 2014.

However, the EGM also identified a need to synthesise this evidence to understand if these interventions effectively address climate change, biodiversity loss and human wellbeing. Thirty‐five systematic reviews (SRs) examined the effects of community‐based or decentralisation of land management or monitoring, land rights, and protected areas (including three SR protocols). Although the number of SRs seems high, 27 of these SRs were assessed as having low confidence, meaning that they are not aligned with international standards for conducting and reporting SRs (The Methods Group of the Campbell Collaboration 2019a, 2019b; Higgins et al. 2022) and, therefore, have a higher risk of being subject to biases when searching, selecting and analysing the evidence. Among the five SRs that were appraised as having high/medium‐confidence, the newest was published more than 5 years ago (Waddington et al. 2019; Geldmann et al. 2013; Pullin et al. 2013; Lawry et al. 2012; Samii et al. 2015). In the context of the rising number of evaluations of these three interventions, these reviews do not reflect the most recent implementation advancements or knowledge of what works.

Moreover, four of these SRs focused on our land interventions individually. Geldmann et al. (2013) focused on the effects of protected areas on habitat cover and species populations, whereas Pullin et al. (2013) were interested in the effects of protected areas on human wellbeing. The mixed methods review of land rights interventions (Lawry et al. 2017) considered implications for environmental themes; however, it included primary studies of land rights interventions without a direct focus on environmental topics. In their review of decentralised forest management, Samii et al. (2015) reported on different types of forest across seven countries. Waddington et al. (2019) focused on two of the three land interventions selected for this review. The authors explored citizen engagement interventions, including community‐based or decentralised land management and monitoring programmes, community‐maintained irrigation programmes, and protected areas.

To understand trade‐offs, potential synergies and the extent to which these interventions can provide triple wins for climate, biodiversity and livelihoods in land systems, we intend to provide an updated SR of the effects of these three land management policies on both environmental and human wellbeing outcomes. This study will synthesise the latest evidence and make its findings accessible to policy‐makers and researchers. This review will help inform future programming decision‐making by highlighting what works across contexts based on the latest and most rigorous evidence. It will also advise researchers on areas where new evaluations are needed to fill evidence gaps. Given the prominence that the nature‐based solutions portfolio is taking to address the climate change and biodiversity crises and the need to contribute to improving prospects for wellbeing (Sandbrook et al. 2023), the knowledge generated by this SR will increase the scope for improved decision‐making and investments in conservation and development (Kinol et al. 2023).

## 2 Objectives

The purpose of this review is to identify, assess and synthesise the evidence of the effects of three land management interventions (community‐based or decentralisation of land management and monitoring; land rights; and protected areas) on environmental and human well‐being outcomes in LMICs. We aimed to answer the following questions:

1. What are the effects of these three interventions on environmental and human well‐being outcomes? Do effects vary by population, location, or other factors?
2. What are the barriers, enablers, and trade‐offs between environmental and human outcomes that impact the effectiveness of the specified interventions?
3. What is the cost‐effectiveness of these interventions?

## 3 Methods

This review will follow best practices for conducting and reporting SRs of effectiveness in international development (The Methods Group of the Campbell Collaboration 2019a, 2019b; Waddington et al. 2012). The review relies on evidence gathered in the CCB EGM produced in 2024 (Marion et al. 2024).^3^ The CCB EGM displays the available rigorous evidence on the effects of climate change and biodiversity interventions on environmental and human well‐being outcomes in LMICs. It includes more than 1605 rigorous IEs and SRs of interventions across four natural systems and productive activities: (i) land and forests; (ii) agriculture and livestock; (iii) aquaculture and fisheries; and (iv) coasts and oceans. A systematic search of studies was conducted in March 2024, followed by the screening and selection of studies from April to June 2024. For research question 1, we will not search or screen for further rigorous evidence. The evidence for research questions 2 and 3 will rely on the programmes rigorously evaluated.

### 3.1 Inclusion Criteria

Based on the CCB EGM, we present the criteria for including studies in the review.

#### 3.1.1 Types of Studies

The review will cover rigorous IEs from the CCB EGM (we will not include SRs). Specifically, we will include studies using experimental and quasi‐experimental designs (the full list of included designs is presented in Appendix S1). These study designs can establish causal relationships between interventions and outcomes if carefully and credibly executed by constructing a counterfactual to address what would have happened in the absence of the intervention (Aloe et al. 2017; Gertler et al. 2011; Reeves et al. 2017). The counterfactual may be inferred from a group that does not receive the intervention (as in a randomised controlled trial), receives an alternative intervention, or may be constructed retrospectively by researchers (as in propensity score matching or interrupted time series designs).

#### 3.1.2 Types of Participants

We will consider studies of the effects of interventions focused on participants residing in LMICs as categorised by the World Bank income classification.^4^ Appendix S2 provides the list of these countries. The classification will reflect the country's income status when the intervention started. Studies analysing more than one country will also be eligible for inclusion if they provide at least one effectiveness estimate specific to LMICs.^5^ We will not exclude studies based on their setting. Hence, we will include evaluations of interventions implemented across rural and urban areas, whether at local, regional, national, or international scales, and those targeting communities, households or individuals.

#### 3.1.3 Types of Interventions

We define an intervention as a deliberate decision or series of actions carried out by an entity – such as a government agency, non‐governmental organisation (NGO), private company, or consortium – with the aim of positively impacting events or outcomes in a replicable way. The three interventions that will be included in this review relate to land management, meaning that they belong to the CCB EGM's *Land and Forests* intervention group and occurred within major types of natural land systems in LMICs, namely (tropical) forests, woodlands, grasslands, savannas, inland wetlands, and peatlands. The list of interventions is detailed in Table 1.

**Table 1:** List of included intervention types.

CCB EGM group	Intervention	Definition
Land and forests	Protected areas	Regulatory framework on protected areas such as national parks or reserves, where access and use of resources is either fully restricted or regulated, as per the International Union for Conservation of Nature categorisation (Dudley 2008). Excludes community‐based management or monitoring interventions.
	Land rights	Formal registration of land, either through the conversion of communal or non‐demarcated rural land to freehold title or the statutory recognition of customary or communal rural land rights. This category also includes other land‐tenure types, such as sustainable‐use protected areas or indigenous‐related tenures (Pacheco and Meyer 2022).
	Community‐based or decentralised land management and monitoring	Interventions that foster community participation in land management decision‐making processes. Decentralisation typically involves transferring some degree of responsibility over land management from central governments to other stakeholders (private sector, local communities, or government). Community participation may take the form of fully decentralised land management, monitoring of external management, or reporting on land use.

#### 3.1.4 Types of Outcome Measures

We will include studies that reported intervention effects on both environmental and human well‐being outcomes, aiming to identify possible trade‐offs, synergies, and unintended consequences of these land management interventions. The review will not cover relevant studies that reported outcomes for only one of these groups. While we have mapped all outcomes covered in the CCB EGM (Table 2 provides the full list of outcomes), we will not exclude other outcomes reported in these evaluations. If identified, we will assess the extent to which these new outcome measures can be incorporated into the analyses.

**Table 2:** List of included outcomes.

CCB EGM group	Sub‐group	Outcome	Definition
Environment	Intermediate outcomes	Natural resource use and management	Measures of land and water use activities undertaken for the purpose of economic production (e.g., cropland, livestock grazing, human settlements), as well as for the maintenance and restoration of environmental functions. Also includes land and water use conservation, activity spillage, and leakage metrics.
		Land and water cover	Measures of the physical land type such as forest, grassland, or open water. Includes metrics such as tree and vegetation density and biomass cover, and area under conservation, protection or restoration measures.
	Biodiversity	Environment status and health	Measures of the condition of any type of land, water, or mixed environments, such as soil health, ocean and freshwater quality.
		Species complexity	Measures of the stock and changes in population size (abundance) and demographic attributes, along with diversity of species within a particular area and seasonal fluctuations (e.g., within‐year variation). Also includes measures of illegal wildlife trade across all applicable natural systems.
		Habitat structural complexity	Measures of habitat structural complexity (e.g., connectivity and lack thereof) and processes (e.g., productivity, trophic chain diversity), that determine ecosystem functions to maintain healthy plant and animal populations and ecosystem processes (e.g., refuge for migratory species).
	Climate mitigation	GHG emissions	Measures of GHG emissions, including changes in amount emitted, avoided, or leaked to another area. May also include measures of GHG intensity per productive unit (e.g., methane intensity in livestock production).
		Carbon storage and sequestration	Measures of carbon stocks and flows in biomass and above and below ground organic matter. Includes both soil organic carbon (temporary sequestration) and permanent carbon elimination measures.
Human well‐being and poverty alleviation	Intermediate outcomes	Knowledge acquisition	Measures of knowledge and formal and informal education, including knowledge of behaviours and technologies related to climate change and biodiversity enhancements and attitudes towards these behaviours and technologies.
		Practice and technology adoption	Measures of adoption and dissemination of practices and technologies related to climate change mitigation, adaptation, and biodiversity protection, conservation, and restoration. Includes measures of the frequency, intensity, and quality of practice implementation. This category also includes input usage efficiency and costs/investments/expenditure in agriculture, livestock, aquaculture and fisheries.
		Land rights and tenure	Measures of individual or household access to land rights or under land reform, titling or registration. This category also includes attitudes and perceptions towards land access.
	Human welfare (to support adaptive capacities)	Productivity	Measures of productivity in land, agriculture, livestock breeding, and aquaculture. Outcomes may include crop yields, land productivity, and livestock by‐product yields (e.g., dairy) in agriculture; pond productivity and fish catches in aquaculture and fisheries.
		Income, assets and basic materials	Measures of household or individual income and proxy indicators such as profit, revenues, assets and expenditures. This category also includes measures of access to basic materials (housing, shelter, fuel, electricity) as well as measures of poverty status, incidence and severity based on income/assets.
		Employment and livelihoods	Measures of individual or household employment status, revenue‐generating activities, income sources (including external sources, such as remittances or transfers), savings, and market access indicators. Also includes measures of livelihoods and productive activity diversification or geographical relocation (linked to economic migration).
		Food security	Measures of food security across the four dimensions included in the Declaration on Food Security (FAO 2009): food availability, access, utilisation, and stability. These are typically measured using a range of indicators such as food consumption and expenditure. This category also includes measures of poverty based on food consumption/expenditure.
		Nutrition	Measures of individual or community nutritional status and food bio‐nutrition characteristics. Measurements include diet nutritional content, vitamin and micronutrient status and deficiencies. Category includes diseases directly caused or strictly associated with nutrient status.
		Health and well‐being	Measures of physical and mental health status, disease exposure and prevalence, and access to health care. Category excludes diseases directly caused or strictly associated with nutrient status.
		Social relations	Measures of interactions between individuals or communities, including conflicts, collective action, trust, and networks.
		Governance and empowerment	Measures of decision‐making, including participation, control, accountability or transparency of decisions in formal or informal institutions and processes. This category also includes measures of empowerment.
		Water access	Measures of clean water availability, disbursement, and reliability for individuals, households or communities.
		Clean air	Measures of access to clean air and air quality indicators.
		Climate risk exposure and resilience	Measures of individual, household, or community exposure to climate shocks (including extreme weather and natural disasters such as floods, droughts, heat waves, wildfires), as well as measures of security, vulnerability and resilience related to climate. This category also includes risk‐sharing measures.
		Multi‐dimensional poverty	Measures of poverty that cover multiple indicators into an index, including, for example, household health, education, assets ownership, access to hot water, type of dwelling, employment status, etc.

#### 3.1.5 Other Inclusion Criteria

- Language: Studies published in any language, although the search terms used in the CCB EGM were in English only.
- Study status: Published or unpublished studies (grey literature), whether completed or ongoing (i.e., prospective study records, protocols, and trial registrations). In the case of ongoing evaluations, we will only include them in the analyses if we identify a completed report before the start of the review.
- Publication date: Studies published in 2000 or later.
- Publication type: Academic and grey literature, including peer‐reviewed journals, working paper series, organisational reports, and unpublished author manuscripts.

### 3.2 Search Methods for Identification of Studies

As this review is based on studies identified in the CCB EGM, we will not search for additional quantitative evidence within academic and grey literature sources, nor perform citation tracking to address research question 1. However, to address research questions 2 and 3, we will search for qualitative and cost‐related evidence.

#### 3.2.1 Searching Other Resources

Using the ‘effectiveness+’ framework (Snilstveit 2012), we will search for qualitative information to identify the barriers and enablers that affect the effectiveness of included interventions. The search for additional cost‐related evidence will aim to inform the interventions' cost‐effectiveness. According to Brown and Tanner (2019), only about 15% of IEs within the broader international development field report intervention cost data, highlighting the need to search for other resources. We will use the following strategies to identify additional documentation from included interventions:

1. Identify descriptive, qualitative, and cost data available in the quantitative IEs included in this review.
2. Conduct backward and forward citation tracking of the quantitative IEs included in this review. Backward citation tracking involves screening studies' reference lists or bibliographies to identify other eligible studies cited within the text. We will perform hand searches and manually record the number of studies screened. Forward citation tracking involves searching for eligible studies that cite the originally included study. We will utilise Google Scholar to track forward citations.
3. Perform a targeted search for additional intervention documents using the names of the interventions included in this review. The search will be conducted on Google (or a similar search engine) and on the websites of the intervention implementers and funders.
4. Contact key researchers and organisations working on issues related to this review. This will include engaging with our expert advisory group to gather suggestions for other relevant documentation of included interventions.

Based on these steps, we will focus on the following types of studies:

- A qualitative study: It should use primary data based on qualitative research methods for data collection and analysis. It should explicitly report the research question, procedures for collecting data, procedures for analysing data, and information on sampling and recruitment. Based on Snilstveit (2012), we will consider studies that report sufficient details of its methodology and methods, including sampling strategy, data collection, and methods of analysis.
- A quantitative descriptive study: It should use descriptive quantitative research methods for primary data collection and analysis. It should explicitly report the research question, procedures for collecting and analysing data, and information on sampling and recruitment.
- A process evaluation: It should assess whether an intervention is being implemented as intended, identify what is working well (or less well), and explore the reasons why. It may include collecting qualitative and quantitative data from different interest holders to address subjective issues (e.g., perceptions of intervention success) and objective issues (e.g., how an intervention was operationalised). It may also collect organisational information.
- A project document: We will collect project documents produced at any stage of the intervention cycle. These documents may describe the background and design of an intervention or the resources available for a project. While these documents may not include a detailed analysis of primary evidence, they should provide facts about interventions. The purpose of including these documents is to ensure sufficient information is available about the context and interventions described in the included studies.

For cost data, we will search for primary studies reporting unit or total costs for intervention implementers, participants or non‐participants. Relevant types of studies include full economic evaluations (e.g., cost–benefit, cost‐effectiveness, and cost‐utility analyses), partial economic evaluations (e.g., cost analyses, cost‐comparison studies, and cost‐outcome descriptions), or any other documentation reporting cost data of the interventions (Shemilt and Mugford 2009).

### 3.3 Data Collection and Analysis

#### 3.3.1 Data Extraction and Management

We will extract the following types of data from each study included in the review, using a standardised data extraction form. The full provisional data extraction form is provided in Appendix S3.

- Descriptive data, such as study authors, publication date, study status, and other characteristics such as country, intervention and outcome types, and intervention design of study's interest.
- Methodological information on study design, analysis method, and type of comparison, if relevant.
- Quantitative data for outcome measures, including descriptive information about the outcomes, sample sizes of the intervention and comparison groups, outcome means and standard deviations, and test statistics (e.g., *t*‐tests, *F*‐tests, *p*‐values, and 95% confidence intervals).

Some of these data has already been coded for the CCB EGM. For the extraction of additional fields, descriptive data and methodological information will be single‐coded by a trained reviewer and checked for agreement by another one. Quantitative data will be coded by two trained reviewers independently, and any disagreement will be resolved through discussion with a third reviewer.

#### 3.3.2 Assessment of Risk of Bias in Included Studies

For quantitative IEs included in this review, we will assess their risk of bias using 3ie's tools (see Appendix S4 for more information). It includes the bias domains and extensions from Cochrane's ROBINS‐I tool and RoB2.0 (Higgins et al. 2016; Sterne et al. 2016). This assessment examines both the internal validity and statistical conclusion validity of experimental and quasi‐experimental IE designs (Waddington et al. 2012). Two reviewers will independently conduct the risk of bias assessment and resolve disagreements with a third reviewer as needed.

Within included studies, we will assess the risk of bias for each estimate based on the following domains by answering whether the estimate is free from each bias, with a response set of ‘Yes’, ‘Probably Yes’, ‘Probably No’, ‘No’, and ‘No Information’:

- Factors relating to baseline confounding and biases arising from differential selection into and out of the study (e.g., assignment mechanism).
- Factors relating to bias due to missing outcome data (e.g., assessment of attrition).
- Factors relating to biases due to deviations from intended interventions (e.g., performance bias and survey effects) and motivation bias (Hawthorne effects).
- Factors relating to biases in outcomes measurement (e.g., social desirability or courtesy bias, recall bias).
- Factors relating to biases in the reporting of analysis.

We will report the assessment results for each of the domains. In addition, we will use the assessment results to produce an overall rating for each study as either ‘High risk of bias’, ‘Some concerns’ or ‘Low risk of bias’, drawing on the decision rules in RoB2.0 (Sterne et al. 2019):

- ‘High risk of bias’: If any of the bias domains were assessed as ‘No’ or ‘Probably No’.
- ‘Some concerns’: If one or several domains were assessed as ‘No Information’ and none were ‘No’ or ‘Probably No’.
- ‘Low risk of bias’: If all the bias domains were assessed as ‘Yes’ or ‘Probably Yes’.

We will describe the reliability of the studies included in our analysis and explore whether differences in estimated effects are linked to varying risk of bias across studies. To test how the risk of bias affects the results, we will perform a sensitivity analysis described in Section 3.3.10.

#### 3.3.3 Measures of Treatment Effect

An effect size (or treatment effect) represents the direction and magnitude of the difference in outcomes between groups of observations, such as the difference between outcomes in intervention and comparison groups (Borenstein et al. 2009; Valentine et al. 2015).

Effect sizes reported in empirical studies, however, are often dependent on the outcome scale or unit used in the study, making them difficult to compare directly across studies. To enable cross‐study comparisons of effect magnitudes, we will extract data from each study and calculate standardised effect sizes. The appropriate formulae for these calculations will be selected based on the data provided in the included studies and the outcome type. Appendix S5 provides the details on the effect size formulae.

When different outcome measures exist within the same outcome category, we will convert estimates to the most common standardised metric to facilitate the comparability of the estimated effect sizes. For this, we will apply the common transformations outlined by Borenstein et al. (2009) to convert between different measures of standardised effects.

#### 3.3.4 Criteria for Determination of Independent Findings

It is crucial that our analysis accurately captures and accounts for the co‐dependencies between study estimates. Standard meta‐analytic methods assume that effect size estimates are independent. However, it can lead to a distorted or exaggerated understanding of the available evidence if failing to qualitatively recognise when estimates are derived from the same intervention.

Dependent effect sizes can arise in several situations. For instance, co‐dependencies between estimates may occur when multiple publications originate from a single study or when several studies use the same data set. Additionally, some studies may have multiple treatment arms that are compared to a single control group. Other studies might report outcome measurements taken at different time points or use multiple outcome measures to assess related outcome constructs. All these scenarios result in statistically dependent effect size estimates (Borenstein et al. 2009).

We will assess the extent of relationships across the studies included in the review. To avoid double‐counting identical evidence, we will link related papers before conducting data analysis. Information provided in the included studies, such as sample sizes, intervention characteristics, and key intervention implementing and funding partners, will be used to support these assessments.

When multiple publications report on the exact same effect, we will select one study for data extraction and extract additional information from linked studies as needed. To identify the main study, priority will be given to journal articles, and in cases involving multiple reports or working papers, the most recent publication will be selected.

We will extract effects reported across different interventions, outcomes and subgroups within each study. To address dependent effect sizes, we will apply data processing and selection techniques. Several criteria will be used to select a single effect estimate per outcome per study. Appendix S6 provides the detailed criteria on effect estimate selection. Alternatively, we may use robust variance estimation (RVE) analyses (Fisher and Tipton 2015; Hedges et al. 2010) to include all available data, even when it is statistically dependent. We may consider this approach when substantively relevant and when we reach the minimum degrees of freedom required to provide valid inferences.

#### 3.3.5 Unit of Analysis Issues

Unit of analysis errors can occur when the unit of allocation for a treatment differs from the unit of analysis for the effect size estimate, and this discrepancy is not accounted for in the analysis (e.g., by clustering standard errors [SEs] at the level of allocation). We will assess the included studies for the prevalence of these issues and, where they are identified, adjust the reported SEs using the following formula (Higgins et al. 2022; Hedges 2009):

$${\left(d\right)}^{\prime }=\left(d\right)\;\cdot \;1+\left(m-1\right)c,$$

where *d* is the effect size, *m* is the average number of observations per cluster, and *c* is the intra‐cluster correlation coefficient (ICC). If the included studies use robust Huber‐White SEs to account for clustering, we will calculate the SE of *d* by dividing by the *t*‐statistic for the coefficient of interest.

We will search the literature for an appropriate ICC value. If no such value is available, we will assume an ICC of 0.05, as described by Waddington et al. (2014).

#### 3.3.6 Dealing With Missing Data

In cases of missing or incomplete data, we will make every effort to contact study authors to obtain the required information. If the necessary data cannot be obtained, we will report the study's characteristics, but note that it could not be included in the meta‐analysis or effect size reporting due to missing data.

Following the recommendations of Mullan et al. (2009) on collating data for SRs, we will document the number of studies for which authors were contacted, the information requested, key details about the method of eliciting information, and the responses received. When relevant, we will also report the impact of the information obtained from authors on the results, including sensitivity analyses.

#### 3.3.7 Data Synthesis

We will conduct meta‐analyses on studies that are sufficiently similar. Studies will be combined in a meta‐analysis if there are at least two effect sizes that share a comparable outcome construct and have similar comparison group conditions, following the approach described by Wilson et al. (2011). Provisionally, we will combine studies in the same analysis if they evaluate either the same type of intervention or the same type of outcome. We will use inverse‐variance weighted, random effects meta‐analytic models to account for heterogeneity across interventions and contexts (Higgins et al. 2022). Meta‐analyses will be conducted using R software (R Core Team 2018), specifically the *metafor* package (Viechtbauer and Cheung 2010) for independent effects and the robumeta package (Fisher et al. 2023) for RVE analyses. If there are too few studies, or if the included studies are too heterogeneous in terms of interventions or outcomes, we will instead provide a narrative discussion of individual effect sizes along the causal chain. Additionally, we will highlight gaps in the evidence and explain why these gaps exist.

#### 3.3.8 Assessment of Reporting Biases

To reduce the possibility of publication bias, we identified and included unpublished studies in the review. We will visually inspect funnel plots for each outcome with at least 10 studies reporting such measures (Higgins et al. 2022). In addition, if a meta‐analysis is feasible, we will test for the presence of publication bias through the use of contour‐enhanced funnel graphs (Peters et al. 2008) and statistical tests (Egger et al. 1997) for outcomes for which we identified at least 10 studies, as suggested by Sterne et al. (2011).

#### 3.3.9 Subgroup Analysis and Investigation of Heterogeneity

In our analysis, we intend to examine and discuss the distribution of estimated effects across intervention and outcome types. We will also statistically assess heterogeneity by calculating the *Q* statistic, *I* ^2^, and *τ* ^2^ to provide an estimate of the amount of variability in the distribution of study effect sizes (Borenstein et al. 2009). We will complement this assessment with a graphical analysis using forest plots and, whenever feasible, we will conduct moderator analyses using meta‐regression analysis to investigate sources of heterogeneity.

Following the PROGRESS‐PLUS approach (Lipsey 2009), we will assess moderators falling into three broad categories of extrinsic, methodological and substantive characteristics. Examples of these categories include:

- Extrinsic characteristics, such as a funder of study (e.g., NGO, private sector, and government investments), publication type, and publication date.
- Methodological characteristics, such as study design, risk of bias, length of follow‐up, and types of outcome measures.
- Substantive characteristics, such as participant characteristics (e.g., gender, age, socio‐economic status, and education), context (e.g., geographical setting), intervention type, intervention features, and type of intervention implementers.

We will use random effects meta‐regression to investigate the association between moderator variables and heterogeneity of treatment effects (Borenstein et al. 2009), and subgroup analyses to investigate heterogeneity by treatment subgroups (e.g., men and women, poor and non‐poor, and so on). If these strategies are not possible (e.g., if we do not have sufficient studies or data), we will discuss and explore the factors that may be driving the heterogeneity of results narratively by conducting cross‐case comparisons (Miles and Huberman 1994).

#### 3.3.10 Sensitivity Analysis

We will conduct a sensitivity analysis to determine whether the results of the meta‐analysis are sensitive to the removal of individual studies. This will involve excluding each study one at a time and assessing how the results change. Additionally, when relevant, we will evaluate the impact of including studies with a high risk of bias by removing these studies from the analysis and comparing the findings to the main meta‐analysis results.

To further examine the robustness of our results, we will assess their sensitivity to potential outliers. Specifically, we will use studentised residuals to identify studies that may have unusually large effect estimates (Viechtbauer and Cheung 2010). Studies with a studentised residual exceeding the 100 × (1 − 0.05/(2 × *k*))th percentile of a standard normal distribution will be considered as potential outliers.

#### 3.3.11 Treatment of Qualitative Evidence

We will extract data from qualitative studies, process evaluations, descriptive quantitative studies, and project documents identified through the search approach outlined in Section 3.2.1. The extracted data will focus on the contexts of implementation, barriers and facilitators affecting intervention effectiveness, and unintended consequences of the interventions. To ensure consistency, we will use a standardised data extraction form, with the complete form provided in Appendix S3. A trained reviewer will perform the initial data extraction, which will then be checked for accuracy and agreement by a second reviewer.

We will assess the quality of the included qualitative studies, process evaluations, and descriptive quantitative studies using a mixed‐methods appraisal tool developed by Langer et al. (2016) and applied by Snilstveit et al. (2018). This tool builds on the Critical Appraisal Skills Programme (CASP) checklist (CASP 2011) and the mixed‐methods appraisal tool developed by Pluye et al. (2011). The critical appraisal tool we will use is presented in Appendix S7.

Using this appraisal tool, we will evaluate the adequacy of reporting, data collection, presentation, analysis, and the conclusions drawn in each study. The quality of the studies will be assessed across six appraisal domains:

1. The defensibility of the applied research design in addressing the research question under investigation.
2. The defensibility of the selected research sample and the process of selecting research participants.
3. The rigour of the technical research conducted, including the transparency of reporting.
4. The rigour of the applied analysis and the credibility of the study's claims, given the nature of the presented data.
5. The consideration of the study's context (for qualitative studies only).
6. The reflexivity of the reported research (for qualitative studies only).

Each appraisal domain will be rated as low, medium, or high trustworthiness. We will determine an overall appraisal rating for a study based on the ratings given to each domain.

For the assessed qualitative studies, process evaluations, and descriptive quantitative studies, we will filter out particularly low‐quality ones at this stage using a fatal flaw approach, as outlined by Dixon‐Woods et al. (2005). For studies that fail to meet either of the above assessment criteria domains from 1 to 4, we will not use their research findings in our synthesis. Their descriptive data, such as details about the applied intervention, will still be reported in this review.

Project documents will not undergo critical appraisal because they primarily provide factual information about interventions, such as their design and available resources. They typically lack extensive analysis of primary evidence but still offer useful details about the interventions. However, we will cross‐check the information reported in these documents (e.g., the intervention name, implementing agency, context, and timeline) to ensure they correspond to the interventions included in our review. For this cross‐check, we will use a triangulation approach by utilising data collected from a range of sources during the additional search, where possible, to enhance confidence in the trustworthiness of information.

To identify implementation and contextual factors that may influence programme success (Thomas et al. 2014), a thematic synthesis of qualitative evidence will be conducted. We will use computer‐aided qualitative data analysis tools, focusing on the following domains:

- Context: Factors beyond the programme's control that affect its impact, such as political unrest, societal norms, economic recessions, or cultural beliefs.
- Intervention design: Variables related to the design and planning of the intervention. This includes the blueprint or schedule of the intervention, typically outlining its components and the sequence of their application (e.g., planning training sizes and modes for participants in community‐based land management).
- Intervention implementation: Variables that arise during the practical application of the intervention are often not anticipated during the planning stage (e.g., participants' attendance or uptake of the programme).
- Population characteristics: Variables related to the target population of the intervention or the population where its effects are measured, if these differ. Examples include socio‐economic status. It is important to distinguish these from sample characteristics, which describe the study sample's composition, and instead focus on how these characteristics might have influenced the intervention's effects.
- Influence on intervention effect: Qualitative data describing how context, intervention design, implementation, and population characteristics functioned as barriers or facilitators to intervention effects. This also includes any unintended results related to these factors, where such information is reported.

#### 3.3.12 Treatment of Cost Data and Economic Evaluations

We will collate and analyse any cost data related to the programmes included for research question 1 (see Section 3.2.1 for details on the search for this evidence). When cost data is available, we will code, appraise the reliability and synthesise this evidence following 3ie's ex‐post cost‐effectiveness analysis approach (Acharya et al. 2024) under the guidance of a technical expert. We will extract cost‐related data, including details of intervention unit costs, total costs, depreciation methods, and year and currency used, from each study included in the review. We will use a standardised data extraction form (presented in Appendix S3).

We will classify the reliability of the extracted information into five ranks based on the accounting methods employed, the source of the cost estimates reported, and the nature of this information. The criteria for each rank are provided in Appendix S8. Cost information will be tabulated and synthesised narratively with descriptions of the different approaches used to derive intervention costs, and effectiveness analysis will be synthesised if a minimum number of studies with similar characteristics allows for it. The cost‐effectiveness analysis will not cover nonsignificant effects (Dhaliwal et al. 2013). If this effect is precisely measured, the intervention has been shown to have no significant effect, so analysing its cost would be irrelevant. If the effect was measured with less precision, uncertainty about the intervention's true effectiveness would make it hard to draw meaningful conclusions about its cost‐effectiveness.

#### 3.3.13 GRADE Analysis for Assessing Certainty of Evidence

We will evaluate and present the certainty of the evidence using an adapted version of the Grading of Recommendations Assessment, Development, and Evaluation (GRADE) system (Guyatt et al. 2008). This adapted version will be tailored to the context of the studies included in this review. We will assess whether the true effect of an intervention, relative to a control group, shows a measurable difference from having no effect for a given outcome (Hultcrantz et al. 2017). This approach focuses on the presence and direction of intervention effects rather than their magnitude. Since many studies will most likely only report the total sample size instead of separate treatment and control group sizes, GRADE tables in this review will be adjusted accordingly to reflect the total sample size. This GRADE analysis will combine results from both quasi‐experimental designs and randomised controlled trials. We will not downgrade the certainty of evidence based on indirectness (i.e., comparing relevant populations, interventions and outcomes of interest; Cuello‐Garcia et al. 2022).

We will present our GRADE ratings in summary of findings tables and provide a narrative description of the ratings, using language approved by the GRADE Working Group (Santesso et al. 2020). We will classify levels of confidence as follows:

- High confidence: It is highly likely that the intervention does (not) have an effect on the outcome of interest.
- Moderate confidence: It is likely that the intervention does (not) have an effect on the outcome of interest.
- Low confidence: It is possible that the intervention does (not) have an effect on the outcome of interest.
- Very low confidence: It is not clear whether the intervention does (not) have an effect on the outcome of interest.

To determine whether the evidence should be downgraded in confidence by one level (serious concern) or two levels (very serious concern), we will consider factors such as limitations in individual studies (risk of bias), inconsistency of results, imprecision, publication bias, the large magnitude of an effect, and whether the evidence is based on a single study.

To complement this assessment of the certainty of the evidence, we will discuss the completeness of the body of evidence to address the research questions based on the data from included studies. We will emphasise the main gaps in the evidence (e.g., related to outcomes understudied, or missing data from studies) and draw implications for generalising the review results.

## Author Contributions

Content: Pierre Marion, Ingunn Storhaug, Sanghwa Lee, Claudia Romero, Constanza Gonzalez Parrao, and Birte Snilstveit. Systematic review methods: Pierre Marion and Constanza Gonzalez Parrao. Statistical analysis: Pierre Marion and Constanza Gonzalez Parrao.

## Conflicts of Interest

The authors declare no conflicts of interest.

## Preliminary Timeframe

Date you plan to submit a draft protocol: December 2024.

Date you plan to submit a draft review: March 2025.

## Plans for Updating This Review

There are no plans for updating the review. The team will explore funding opportunities for updates and extensions of this review upon submission of the final report and as relevant.

## Peer Review

The peer review history for this article is available at: https://www.webofscience.com/api/gateway/wos/peer-review/10.1002/cl2.70062.

## Supporting information


**Appendix 1:** Included study designs. **Appendix 2:** List of included low‐ and middle‐income countries. **Appendix 3:** Provisional data extraction form. **Appendix 4:** Risk of bias assessment tools. **Appendix 5:** Calculating standardised effects. **Appendix 6:** Criteria determining selection of effect estimates for data extraction. **Appendix 7:** Critical appraisal tool for qualitative studies. **Appendix 8:** Reliability classification tool for cost evidence.

## Data Availability

Upon completion of the review, the data and analysis codes used will be publicly available in 3ie's Dataverse.

## Endnotes

1 The ‘tragedy of the commons’ describes a situation where open access resources are progressively depleted as users act in their interest without concern for over‐exploitation of these resources and effects on other users (Hardin 1968).

2 These assumptions are not meant to be exhaustive but are provided as examples. When reviewing the evidence, we identify and explore other assumptions as relevant.

3 The online map is available at: https://developmentevidence.3ieimpact.org/egm/ccb-egm. The EGM report provides detailed information on the search strategy, search results, inclusion criteria used and screening and extraction methods and results.

4 Available at: https://datahelpdesk.worldbank.org/knowledgebase/articles/906519-world-bank-country-and-lending-groups.

5 We will also include studies from countries that have had a high‐income status for only 1 year before reverting to L&MIC status. At the time of writing this protocol, this exception only applies to Argentina (2014, 2017), Venezuela (2014), Mauritius (2019), and Romania (2019).
