# Supplementary material for: PROTOCOL: The Effects of Land Management Policies on the Environment and People in Low‐ and Middle‐Income Countries: A Systematic Review
Source: Campbell Syst Rev. 2025 Oct 27;21(4):e70062. doi: 10.1002/cl2.70062 (PMC12558594; doi:10.1002/cl2.70062)
Supplement: Supplementary file 1 — Appendix 1: Included study designs. Appendix 2: List of included low‐ and middle‐income countries. Appendix 3: Provisional data extraction form. Appendix 4: Risk of bias assessment tools. Appendix 5: Calculating standardised effects. Appendix 6: Criteria determining selection of effect estimates for data extraction. Appendix 7: Critical appraisal tool for qualitative studies. Appendix 8: Reliability classification tool for cost evidence. [file CL2-21-e70062-s001.docx]

**PROTOCOL: The effects of land management policies on the environment and people in low- and middle-income countries: A systematic review**

Appendices

​**Table of Contents**

[Appendix 1. Included study designs 3](#_Toc185444864)

[Appendix 2. List of included low- and middle-income countries 5](#_Toc185444865)

[Appendix 3. Provisional data extraction form 8](#_Toc185444866)

[Appendix 4. Risk of bias assessment tools 12](#_Toc185444867)

[Appendix 5. Calculating standardised effects 40](#_Toc185444868)

[Appendix 6. Criteria determining selection of effect estimates for data extraction 43](#_Toc185444869)

[Appendix 7. Critical appraisal tool for qualitative studies 45](#_Toc185444870)

[Appendix 8. Reliability classification tool for cost evidence 51](#_Toc185444871)

[Appendix References 54](#_Toc185444872)

# Appendix 1. Included study designs

We will include studies using experimental and quasi-experimental study designs to measure a change in outcomes that is attributable to an intervention. This includes studies that apply one of the following approaches:

1. Randomised controlled trials (RCTs), with assignment at individual, household, community, or other cluster level, and quasi-RCTs using prospective methods of assignment (such as alternation)
2. Natural experiments with clearly defined intervention and comparison groups, which exploit natural randomness in implementation assignment by decision makers (e.g., public lottery) or random errors in implementation
3. Regression discontinuity designs (RDD) or fuzzy-RDD
4. Instrumental variables (IV)
5. Endogenous treatment-effects models, endogenous switching regression, and other methods synonymous to the Heckman two-step model.
6. Difference-in-differences (DID), two-way fixed-effects (TWFE), and two-way Mundlak regressions (TWM).
7. DiD models will include an interaction term between a time and intervention variable in a regression model. They may also regress an intervention variable on an outcome variable measuring the changes in outcomes over time or present a *t*-test comparing changes in outcomes over time between the intervention and control group.
8. TWFE regressions must include time fixed-effects and unit fixed-effects at the level of the intervention (or lower). For example, if the intervention varies at a village level, it must include either village fixed-effects or fixed-effects of a smaller unit, such as households.
9. TWM models should be synonymous with the approach described by Wooldridge (2021). This includes correlated random-effects and pooled OLS regression models that control for unit-specific time averages and time-period specific cross-sectional averages.
10. Interrupted time series (ITS) models, with or without a contemporaneous comparison group. This includes segmented regressions, where the time-period is divided into pre- and post-intervention segments, and separate intercepts and/or slopes are estimated for each segment.
11. Weighting and matching approaches that control for observable confounding, including non-parametric approaches (e.g., statistical matching, covariate matching, coarsened-exact matching, propensity score matching) and parametric approaches (e.g., propensity-weighted multiple regression analysis).
12. The synthetic control method

# Appendix 2. List of included low- and middle-income countries

| **Low- and middle-income countries (L&MICs)** | | | |
| --- | --- | --- | --- |
| Afghanistan | Dominican Rep. | Liberia | Serbia |
| Albania | Ecuador | Libya | Sierra Leone |
| Algeria | Egypt, AR | Macedonia, FYR | Solomon Islands |
| Angola | El Salvador | Madagascar | Somalia |
| Armenia | Eritrea | Malawi | South Africa |
| Azerbaijan | Ethiopia | Malaysia | South Sudan |
| Bangladesh | Fiji | Maldives | Sri Lanka |
| Belarus | Gabon | Mali | St. Lucia |
| Belize | Gambia, The | Marshall Islands | St. Vin. & Gren. |
| Benin | Georgia | Mauritania | Sudan |
| Bhutan | Ghana | Mexico | Suriname |
| Bolivia | Grenada | Micronesia, FS | Swaziland |
| Bosnia & Herzegovina | Guatemala | Moldova | Syrian Arab Rep. |
| Botswana | Guinea | Mongolia | Tajikistan |
| Brazil | Guinea-Bissau | Montenegro | Tanzania |
| Bulgaria | Guyana | Morocco | Thailand |
| Burkina Faso | Haiti | Mozambique | Timor-Leste |
| Burundi | Honduras | Myanmar | Togo |
| Cambodia | India | Namibia | Tonga |
| Cameroon | Indonesia | Nauru | Tunisia |
| Cape (Cabo) Verde | Iran, I.S. | Nepal | Turkey |
| Central African Rep. | Iraq | Nicaragua | Turkmenistan |
| Chad | Jamaica | Niger | Tuvalu |
| China | Jordan | Nigeria | Uganda |
| Colombia | Kazakhstan | Pakistan | Ukraine |
| Comoros | Kenya | Pap. New Guinea | Uzbekistan |
| Congo, DR | Kiribati | Paraguay | Vanuatu |
| Congo, Republic | Korea, Dem. Republic | Peru | Vietnam |
| Costa Rica | Kosovo | Philippines | W. Bank & Gaza |
| Côte d'Ivoire | Kyrgyz, Republic | Rwanda | Yemen, Republic |
| Cuba | Lao PDR | Samoa | Zambia |
| Djibouti | Lebanon | São Tomé & Prin. | Zimbabwe |
| Dominica | Lesotho | Senegal |  |

| **Former low- and middle-income countries** | | | |
| --- | --- | --- | --- |
| Czechoslovakia | Mayotte (High income: 1990) | Serbia and Mont | Yugoslavia |
| Gibraltar (High income: 2009-2010) | Netherlands Antilles (High income: 1994-2009) | USSR |  |

| **Transitional countries** | | | |
| --- | --- | --- | --- |
| **Name** | | **L&MIC period** | **High-income country period** |
| American Samoa | 1990-present | | 1987-1989 |
| Antigua and Barbuda | 1987-2001; 2003-2004; 2009-2011 | | 2002; 2005-08; 2012-present |
| Argentina | 1987-2013; 2015-16; 2018-present | | 2014; 2017 |
| Aruba | 1991-1993 | | 1987-1990; 1994-present |
| Bahrain | 1990-2000 | | 1987-1989; 2001-present |
| Barbados | 1987-1988;1990-99; 2001;2003-05 | | 1989; 2000; 2002; 2006-pres |
| Chile | 1987-2011 | | 2012-present |
| Croatia | 1992-2007; 2016 | | 2008-2015; 2017-present |
| Cyprus | 1987 | | 1988-present |
| Czech Republic | 1992-2005 | | 2006-present |
| Equatorial Guinea | 1987-2006; 2015-present | | 2007-2014 |
| Estonia | 1991-2005 | | 2006-present |
| Guam | 1990-1994 | | 1987-1989; 1995-present |
| Greece | 1987-1995 | | 1996-present |
| Hungary | 1987-2006; 2012-2013 | | 2007-2011; 2014-present |
| Isle of Man | 1990-2001 | | 1987-1989; 2002-present |
| Latvia | 1991-2008; 2010-2011 | | 2009; 2012-present |
| Lithuania | 1991-2011 | | 2012-present |
| Macao (SAR) | 1987-1993 | | 1994-present |
| Malta | 1987-1988; 1990-1997;1999; 2001 | | 1989; 1998; 2000; 2002-pres. |
| Mauritius | 1987-2018; 2020-present | | 2019 |
| Nauru | 2016-2018 | | 2015; 2019-present |
| New Caledonia | 1987-1994 | | 1995-present |
| Northern Mariana Islands | 1992-1994; 2002-2006 | | 1995-2001; 2007-present |
| Oman | 1987-2006 | | 2007-present |
| Palau | 1987-2015 | | 2016-present |
| Panama | 1987-2016; 2020-present | | 2017-2019 |
| Poland | 1987-2008 | | 2009-present |
| Portugal | 1987-1993 | | 1994-present |
| Puerto Rico | 1987-1988; 1990-2001 | | 1989; 2002-present |
| Republic of Korea | 1987-1994; 1998-2000 | | 1995-1997; 2001-present |
| Romania | 1987-2018; 2020-present | | 2019 |
| Russia | 1991-2011; 2015-present | | 2012-2014 |
| Seychelles | 1987-2013 | | 2014-present |
| Slovak Republic | 1992-2006 | | 2007-present |
| Slovenia | 1992-1996 | | 1997-present |
| Saudi Arabia | 1990-2003 | | 1987-1989; 2004-present |
| St. Kitts and Nevis | 1987-2010 | | 2011-present |
| Trinidad and Tobago | 1987-2005 | | 2006-present |
| Uruguay | 1987-2011 | | 2012-present |
| Venezuela | 1987-2013; 2015-present | | 2014 |

| **High-income countries** | | | |
| --- | --- | --- | --- |
| Andorra | Faeroe Islands | Kuwait | St. Martin- French |
| Australia | Finland | Liechtenstein | Sweden |
| Austria | France | Luxembourg | Switzerland |
| Bahamas | French Polynesia | Monaco | Taiwan |
| Belgium | Germany | Netherlands | Turks & Caicos Isl |
| Bermuda | Greenland | New Zealand | Untd Arab Emir. |
| Brunei Darussalam | Hong Kong (SAR) | Norway | United Kingdom |
| Canada | Iceland | Qatar | United States |
| Cayman Islands | Ireland | San Marino | Virgin Islands- US |
| Channel Islands | Israel | Singapore |  |
| Curacao | Italy | St. Martin (Dutch) |  |
| Denmark | Japan | Spain |  |

# Appendix 3. Provisional data extraction form

| **Variable group** | **Variable** | **Description** | | | |
| --- | --- | --- | --- | --- | --- |
| Publication Information | Study ID | The unique ID code that is assigned to each included study | | | |
|  | Estimate ID | The unique ID code that assigned to each individual estimate | | | |
|  | Study status | Select one of the following:  i) Completed; ii) Protocol; iii) Ongoing | | | |
|  | Author Name | Authors last names [Open Answer] | | | |
|  | Year of Publication | Year published (publication date, not preprint or first online publication dates) | | | |
| Intervention Information | Intervention code | Choose one or more intervention code(s) for each corresponding effect size:  i) Protected areas; ii) Land rights; iii) Community-based (or decentralised) land management and monitoring | | | |
|  | Country | Country of intervention | | | |
|  | Exposure to intervention (in months) | For how long are the observations exposed to the intervention? | | | |
|  | Evaluation period (in months) | The total number of months elapsed between the end of an intervention and the point at which an outcome measure is taken post intervention, or as a follow-up measurement.  If less than one month, use decimals (e.g., measurement immediately after the intervention end would be coded as 0, one week would be .25, etc.) | | | |
|  | Intervention description | Provide detailed description of the intervention and its different components such that a reader could easily understand what happened. Include page numbers for quick reference. If two or more interventions are being evaluated, please provide descriptions for each intervention arm under separate rows. | | | |
|  | Cost | Report any cost data provided or comments on cost effectiveness, include the authors' comments on cost data even if quantifications are not provided. Provide details of what the cost relates to or how they have been calculated if possible. Include any information identified from cited documents or linked studies. | | | |
| Method  information | Evaluation  Design | Select one of the options below:  1. Experimental (defined as prospective randomised assignment, where randomisation is implemented by researchers (or by decision makers in the context of an evaluation study)  2. Quasi-experimental (including natural experiments and non-randomised studies). | | | |
|  | Evaluation  Method | If Experimental, then select:  Randomised controlled trial  If Quasi-experiment or natural experiment, then select:  Natural experiment in which exposure to treatment is random  Regression Discontinuity Design (RDD)  Difference-in-Differences (DID) / Fixed effects estimation  Instrumental variable (IV) estimation  Endogenous treatment-effects models (including endogenous switching regression, and other methods synonymous to the Heckman two step model)  Statistical matching (includes PSM or statistical weighting)  Interrupted time series (ITS)  Synthetic controls | | | |
|  | Additional Methods | Select additional method if any. If none, select not applicable. [Open Answer] | | | |
| Estimate Information | Analysis type for this effect size | Free text, what type of analysis was used (Regression, 2SLS, ANCOVA, etc.) | | |  |
|  | Estimate Type | Type of data for this effect size: 1 = Continuous - means and SDs, 2 = Continuous - mean difference and SD, 3 = Dichotomous outcome - proportions, 4 = Regression data - dichotomous outcome, 5 = Regression data - continuous outcome | | |  |
|  | Treatment Effect | 1=Intention to Treat (ITT), 2=Average Treatment Effect on the Treated (ATET), 3=Average Treatment Effect (ATE) 4 = Local Average Treatment Effect (LATE) | | |  |
|  | Unit of analysis | What is the unit of analysis? UOA for this effect size: 1= Individual, 2= Household, 3= Group (e.g., community organisation), 4= Village, 5 = Other, 6 = Not clear | | |  |
|  | Source | Note the page number, table number, column, and row you used to extract the data  [Open Answer] | | |  |
| Treatment variable information | Treatment | Record the treatment variable as written in the model (e.g., the variable name the author uses, such as ("Intervention x Time")  [Open Answer] | | |  |
|  | Treatment type | Describe the types of treatment variable used: i) binary; ii) continuous; iii) categorical; iv) other | | |  |
|  | Comparison | 1=No intervention (service delivery as usual), 2=Other intervention, 3=Pipeline (waitlist) control (still service delivery as usual) | | |  |
|  | Describe Comparison Group | Describe the comparison group [Open Answer] | | |  |
|  | Subgroup | Is this analysis of a subgroup or estimating heterogeneous effects?  0=no, 1=yes | | |  |
|  | Subgroup information | Describe the subgroup or variable interacted with the treatment variable if applicable (e.g., boys, girls).  If no subgroup, select not applicable [Open Answer] | | |  |
| Outcome  Information | Outcome description | Record the outcome for the corresponding effect size. Use this open answer field to enter, in the author’s own words, a description of the outcome. Be selective and concise with the excerpts being transcribed here as to ensure accurate and precise descriptions of the outcome. To the extent possible, be sure to include numbers, units, population, and comparators. Include page numbers with every excerpt extracted. | | |  |
|  | Outcome codes | Choose an outcome code for each corresponding effect size:   - 1. Environmental intermediary outcomes;   2. Climate mitigation outcomes;   3. Biodiversity outcomes;   4. Human intermediary outcomes;   5. Human welfare outcomes | | |  |
|  | Outcome sub-group | Choose an outcome sub-group code for each corresponding effect size:   - 1. Environmental intermediary outcomes: Natural resource use and management; Land and water cover   2. Climate mitigation outcomes; Environmental status and health; Species complexity; Habitat structural complexity   3. Biodiversity outcomes: GHG emissions; Carbon storage and sequestration   4. Human intermediary outcomes; Knowledge acquisition; Practice and technology adoption; Land rights and tenure   5. Human welfare outcomes: Productivity; Income, assets and basic materials; Employment and livelihoods; Food security; Nutrition; Health and wellbeing; Social relations; Governance and empowerment; Water access; Clean air; Climate risk exposure and resilience; Multi-dimensional poverty |  |  |  |
|  | Outcome description | Record the outcome for the corresponding effect size. Use this open answer field to enter, in the author’s own words, a description of the outcome. Be selective and concise with the excerpts being transcribed here as to ensure accurate and precise descriptions of the outcome. To the extent possible, be sure to include numbers, units, population, and comparators. Include page numbers with every excerpt extracted. |  |  |  |
|  | Post-intervention or change from baseline? | 0 = Post-intervention, 1 = Change from baseline |  |  |  |
| Estimate data | Mean treatment | Outcome mean for the treatment group | |  |  |
|  | SD treatment | Outcome standard deviation for treatment group | |  |  |
|  | Mean Control | Outcome mean for the comparison group | |  |  |
|  | SD Control | Outcome standard deviation for control group | |  |  |
|  | Mean difference | Overall mean difference (treatment - control) | |  |  |
|  | SE difference | Standard error of the overall mean difference | |  |  |
|  | Tstat difference | t-statistic of mean difference | |  |  |
|  | p-value difference | p-value of mean difference | |  |  |
|  | Odds ratio | Odds ratio reported in the study | |  |  |
|  | SE odds ratio | Odds ratio standard error reported in the study | |  |  |
|  | Risk ratio | Risk ratio reported in study | |  |  |
|  | SE risk ratio | Risk ratio standard error | |  |  |
|  | Coeff reg | Report the regression coefficient of the treatment effect | |  |  |
|  | SE reg | Report the associated standard error of the regression coefficient. | |  |  |
|  | Tstat reg | Report the associated t statistic of the effect size (coefficient/SE) | |  |  |
|  | CI_LB reg | Report the associated Lower bound of the 95% Confidence interval of the effect size. If CI is reported for a different confidence level, indicate that in the notes section. | |  |  |
|  | CI_UP reg | Report the associated Upper bound of the 95% Confidence interval of the effect size. If CI is reported for a different confidence level, indicate that in the notes section. | |  |  |
|  | P value exact | Exact p value if given, if not, record as written in the manuscript (e.g., p < .001, or p > .05) | |  |  |
|  | Clusters treatment | Number of clusters - treatment group | |  |  |
|  | Clusters control | Number of clusters -  control group | |  |  |
|  | Clusters total | Number of clusters - total sample | |  |  |
|  | N treatment | Sample size - treatment group | |  |  |
|  | N control | Sample size - control group | |  |  |
|  | N total | Sample size - total sample | |  |  |
|  | periods (1 if cross sectional) | Record how many time-period there are in the evaluation (e.g., cross section is 1, panel data with 3 measurements is 3) | |  |  |
|  | Does the sample size need to be corrected? | Often in panel data, models will report number of observations rather than number of participants. In this column you will indicate 1="Yes" if the sample size needs to be divided by the number of periods, and 0="No" if either it is cross-sectional data, or if the authors have already divided the number of observations by the number of panel assessments and thus no correction is necessary. | |  |  |

# Appendix 4. Risk of bias assessment tools

## Tool for randomised experiments

| Question | Coding format | Criteria | Decision rule |
| --- | --- | --- | --- |
| General | | | |
| Study ID | EPPI ID |  |  |
| Estimate ID | EPPI ID Estimate # |  |  |
| Study first author | Open answer |  |  |
| Time taken to complete assessment | Minutes |  |  |
| Design type: What type of study design is used? | 1= Randomised controlled trial (RCT) (random assignment to households/individuals) or quasi-RCT 2= Cluster-RCT (quasi-RCT) |  |  |
| Methods used for analysis: Which methods are used to control for selection bias and confounding? | 1 = Statistical matching (PSM, CEM, covariate matching) 2 = Difference in differences (DID) estimation methods + Fixed effect 3 = IV-regression (2-stage least squares or bivariate probit) 4 = Heckman selection model 5 = Covariate adjusted estimation / cross-section 6 = Propensity weighted regression 7 = Comparison of means 8 = Other (please state) |  |  |
| Design and analysis method description | Open answer | Briefly describe the study design and analysis method undertaken by the authors. Take content from QEX. | |
| Study registration | Open answer | Provide link to pre-analysis plan if available. | |
| 1: Assignment mechanism - Assessment | | | |
| Assignment mechanism: Was the allocation or identification mechanism random or as good as random? | 1= Yes,  2 = Probably Yes,  3 = Probably No,  4 = No,  8 = Unclear | a) The authors describe a random component in sequence generation/ randomization method (e.g. lottery, coin toss, random number generator) and assignment is performed for all units at the start of the study centrally or using a method concealed from participants and intervention delivery. b) If public lottery is used for the sequence generation, authors provide detail on the exact settings and participants attending the lottery. c) If a special randomization procedure is used to ensure balance, it is well described and justified given the study setting (stratification, pairwise matching, unique random draw, multiple random draws etc.). d) A balance table is reported suggesting that allocation was random between all groups including subgroup receiving different treatment within control or treatment groups (if the comparison is relevant for this assessment). | Score “Yes” if all criterion a), b), c) and d) are satisfied. Score "Probably Yes" if only criterion a) and b) are not satisfied OR if only criteria c) is not satisfied. Score “Unclear” if d) is not satisfied because no balance table is reported. Score "Probably No" if d) is not satisfied because there is no balance table reported and there is evidence suggesting a problem in the randomization, such as baseline coefficients in a diff-in-diff regression table are very different or sample size is too small for the procedure used (using stratification when there are less than two units for each intervention and control group in each strata can lead to imbalance). Score “No” if d) is not satisfied because there are large imbalances concerning a large number of variables, providing evidence that the assignment was not random. If this is scored as no, use the NRS tool. |
| Assignment justification | Open answer. Your answer should include all points here (a, b, c), the justification for each point (cite parts from the study) and a page number. a, Yes as... p. XX b No as...p. XX c Yes as... p. XX | Justification for coding decision (Include a brief summary of justification for rating, mentioning your response to all sub questions, cite relevant pages). | |
| 2: Unit of analysis - Assessment | | | |
| Unit of analysis: Is unit of analysis in cluster allocation addressed in standard error calculation ? | 1=Yes 2=No 3=Not reported/unclear 4=Not applicable |  | Score "Yes" if Unit of analysis (UoA) = Unit of randomization (UoR) OR if UoA ≠ UoR and standard errors are clustered at the UoR level OR data is collapsed to the UoR level Score "Not reported/unclear" if not enough information is provided on the way the standard errors were calculated or what the unit of analysis is. Score "Not applicable" if it is not a cluster RCT. Score "No" otherwise. |
| Method used to address differences between UoA and unit of data collection | Open answer |  | |
| 3: Selection bias – Assessment | | | |
| Selection bias: Was any differential selection into or out of the study (attrition bias) adequately resolved? | 1= Yes,  2 = Probably Yes,  3 = Probably No,  4 = No,  8 = Unclear |  | Score "Yes" if there is no attrition or attrition falls into the green zone (see figure A.1 below) and the study establishes that attrition is randomly distributed (e.g. by presenting balance by key characteristics across groups) AND if survey respondents were randomly sampled. Score "Probably yes" if attrition falls into the green zone AND if survey respondents were randomly sampled. Score "Unclear" if there is an attrition problem but no information provided on the relationship between attrition and treatment status, OR if there is not enough information on how the population surveyed was sampled. Score "Probably no" if there is attrition which is likely to be related to the intervention OR there is some indication that the survey respondents were purposely sampled in a way that might have led the sampling to be different between treatment and control groups, or attrition falls into the yellow zone. Score "No" if attrition falls into the red zone. USE ATTRITION GRAPH TAB TO DETERMINE THE ATTRITION ZONE |
| Selection bias justification | Open answer | Justification for coding decision (Include a brief summary of justification for rating, mentioning your response to all sub questions, cite relevant pages). | |
| 4: Confounding – Assessment | | | |
| Confounding and group equivalence: Was the method of analysis executed adequately to ensure comparability of groups throughout the study and prevent confounding | 1= Yes,  2 = Probably Yes,  3 = Probably No,  4 = No,  8 = Unclear | a) Baseline characteristics are similar in magnitude; b) Unbalanced covariates at the individual and cluster level are controlled in adjusted analysis; c) Adjustments to the randomization were taken into account in the analysis (stratum fixed effects, pairwise matching variables)? (Bruhn and McKenzie 2009) | Score “Yes” if criterion a) and b) are satisfied; Score "Probably yes" if a) is not satisfied but b) is satisfied and imbalances are small in magnitude OR if only a) is satisfied.  Score “Unclear” if no balance table is provided or if imbalances are controlled for but they are very large in magnitude and assignment mechanism is not coded as "Yes" or "Probably yes" Score "Probably no" if a) and b) are not satisfied and the magnitude of imbalances are small Score “No” if a) and b) are not satisfied and the magnitude of imbalances are large and covariates are clear determinant of the outcomes. |
| Confounding justification | Open answer | Justification for coding decision (Include a brief summary of justification for rating, mentioning your response to all sub questions, cite relevant pages). | |
| 5: Deviations from intended interventions – Assessment | | | |
| Deviations from intended interventions: Spill-overs, cross-overs and contamination: was the study adequately protected against spill-overs, cross-overs and contamination? | 1= Yes,  2 = Probably Yes,  3 = Probably No,  4 = No,  8 = Unclear | a) There was no implementation issues that might have led the control participants to receive the treatment (implementer's mistake). b) The intervention is unlikely to spill-over to comparisons (e.g. participants and non-participants are geographically and/or socially separated from one another and general equilibrium effects are not likely) or the potential effects of spill overs were measured (e.g. variation in the % of unit within a cluster receiving the treatment). c) There is no risk of contamination by external programmes: the treatment and comparisons are isolated from other interventions which might explain changes in outcomes.  d) There is nothing in the surveys that might have given the control participants an idea of what the other group might receive OR they did but there is no risk that this has changed their behaviors; AND the survey process did not reveal information to the control group that they did not have before (e.g. the study aims to measure increase in take up of a service or product that participants might not know about) Authors might put something in place in the design of the study that allows to control for that survey effect (e.g. a pure control with no monitoring except baseline end line) | Score “Yes” if criterion a), b), c) and d) are satisfied; Score "Probably yes" if there is no obvious problem but there is no information reported on potential risks related to spill overs, contamination, or survey effects in the control group OR if there were issues with spill-overs but they were controlled for or measured. Score “Unclear” if spill-overs, cross-overs, survey effects and/or contamination are not addressed clearly. Score "Probably no" if any of the criterion a), b), c) or d) are not satisfied but the scale of the issue is not clear. Score “No” if any of the criterion a), b), c) or d) are not satisfied and happened at a large scale in the study. |
| Deviations justification | Open answer. Your answer should include all points here (a, b, c), the justification for each point (cite parts from the study) and a page number. a, Yes as... p. XX b No as...p. XX c Yes as... p. XX | Justification for coding decision (Include a brief summary of justification for rating, mentioning your response to all sub questions, cite relevant pages).  For example, intervention groups are geographically separated, authors use intention to treat estimation or instrumental variables to account for non-adherence, and survey questions are not likely to expose individuals in the control group to information about desirable behaviors (‘survey effects’). | |
| 6. Performance bias - Assessment | | | |
| Performance bias: Was the process of monitoring individuals unlikely to introduce motivation bias among participants? | 1= Yes,  2 = Probably Yes,  3 = Probably No,  4 = No,  8 = Unclear | a) The authors state explicitly that the process of monitoring the intervention and outcome measurement is blinded and conducted in the same frequency for treatment and control groups, or argue convincingly why it is not likely that being monitored could affect the performance of participants in treatment and comparison groups in different ways (such as resulting in Hawthorne or John Henry effects).  b) The outcome is based on data collected in the context of a survey, and not associated with a particular intervention trial, or data are collected from administrative records or in the context of a retrospective (ex post) evaluation. | Score “Yes” if either criterion a) or b) are satisfied; Score "Probably yes" if the study is based on data collected during a trial and there is no obvious issue with the monitoring processes but authors do not mention potential risks. Score “Unclear” if it is not clear whether the authors use an appropriate method to prevent Hawthorne and John Henry Effects (e.g. blinding of outcomes and, or enumerators, other methods to ensure consistent monitoring across groups). Hawthorne effects may result where participants know that they are being observed and John Henry Effects may result from participant knowledge of being compared. Score "Probably no" if there was imbalance in the frequency of monitoring in intervention groups, which might have influenced participants' behaviors. Score "No" if neither criterion a) or b) are satisfied. |
| Performance bias justification | Open answer. Your answer should include all points here (a, b, c), the justification for each point (cite parts from the study) and a page number. a, Yes as... p. XX b No as...p. XX c Yes as... p. XX | Justification for coding decision (Include a brief summary of justification for rating, mentioning your response to all sub questions, cite relevant pages). | |
| 7. Outcome measurement bias - Assessment | | | |
| Outcome measurement bias: Was the study free from biases in outcome measurement? | 1= Yes,  2 = Probably Yes,  3 = Probably No,  4 = No,  8 = Unclear | a) Outcome assessors are blinded or the outcome measures are not likely to be biased by their judgement.  b) For self-reported outcomes: respondents in the intervention group are not more likely to have accurate answers due to recall bias; c) For self-reported outcomes: respondents do not have incentives to over/under report something related to their performance or actions, OR researchers put in place mechanisms to reduce the risk of reporting bias (researchers not strongly involved in the implementation of the programme and it is clear that their answers to the survey will not affect what they receive in the future) OR authors have measured the risks of bias through falsification tests or measuring the effect on placebo outcomes in cases where there was a risk of reporting bias. d) Timing issue: the data collection period did not differ between intervention and comparison group, the baseline data is not likely to be affected by the beginning of the intervention or affects a small percentage of the study participants. | Score “Yes” if criterion a), b), c) and d) are satisfied: Score "Probably yes" if there is a small risk related to any of a), b), c) or d) and there is no more information provided to justify the absence of bias OR if there was a high risk of bias but authors have either controlled it in their design or measured it with a placebo outcomes. Score “Unclear” if it there is a high risk related to any of a), b), c) or d) and there is no more information provided to justify the absence of bias.  Score "Probably no" if there are high risk related to a), b), c) or d) and it is clear that authors were not able to control for this bias. Score “No” if there is evidence of bias. |
| Outcome measurement justification | Open answer. Your answer should include all points here (a, b, c), the justification for each point (cite parts from the study) and a page number. a, Yes as... p. XX b No as...p. XX c Yes as... p. XX | Justification for coding decision (Include a brief summary of justification for rating, mentioning your response to all sub questions, cite relevant pages). | |
| 8. Reporting bias - Assessment | | | |
| Analysis reporting: Was the study free from selective analysis reporting? | 1= Yes,  2 = Probably Yes,  3 = Probably No,  4 = No,  8 = Unclear | a) A pre-analysis plan or trial protocol is published and referred to or the trial was pre-registered or the outcomes were pre-registered; b) Authors report results corresponding to the outcomes announced in the pre-analysis plan. If PAP is not available, the authors report results corresponding to the methods section (there is no evidence of outcome reporting bias); c) Authors report results of unadjusted analysis and intention to treat (ITT) estimation, alongside any adjusted and treatment-on-the-treated/complier-average-causal-effects analysis.) d) Authors use the appropriate analysis method (use baseline data when available) and different treatment arms are differentiated in the analysis e) Authors have reported all the analysis which could help understand the results and no other bias is assessed as unclear due to the lack of an important analysis (e.g. a balance table or a subgroup analysis) | Score "Yes" if all the criterion a), b), c), d), and e) are satisfied; Score "Probably yes" if all the conditions are met except a), or if all the conditions are met but there is some element missing that could have helped understand the results better (e); Score "Unclear" if there is not enough information to determine that there is an analysis missing; Score "Probably no" if any of the criterion b), c) or d) are not satisfied; Score "No" if any of the criterion b), c) or d) are not satisfied and there is evidence that the analysis results would be different because large imbalances were not controlled for, compliance was very low and ITT estimation was not reported or different treatment arms were pooled. |
| Analysis reporting justification | Open answer. Your answer should include all points here (a, b, c), the justification for each point (cite parts from the study) and a page number. a, Yes as... p. XX b No as...p. XX c Yes as... p. XX | Justification for coding decision (Include a brief summary of justification for rating, mentioning your response to all sub questions, cite relevant pages). | |
| 9: Other bias - Assessment | | | |
| Other risks of bias: Was the study free from other sources of bias? | 1= Yes,  4 = No | Score “Yes” if the reported results do not suggest any other sources of bias.  Score “No” if other potential threats to validity are present, and note these here (e.g. coherence of results, survey instruments used are not reported) | |
| Other risks of bias - Justification | Open answer | Justification for coding decision  (Include a brief summary of justification for rating, mentioning your response to all sub questions, cite relevant pages). | |
| 10: Blinding - Assessment | | | |
| For observers – Did the study blind outcome assessors? | 1=Yes,  2=No,  8=unclear,  9= N/A | If there is no information, code NO. If there is information but it is ambiguous, code UNCLEAR. | |
| For analysts – Did the study blind data analysts? | 1=Yes, 2=No,  8=unclear,  9= N/A | If there is no information, code NO. If there is information but it is ambiguous, code UNCLEAR. | |
| Blinding method(s) used to blind | Open answer (including describe method of placebo control)No 9= N/A | Describe method(s) used to blind | |
| 8: External validity | | | |
| External validity | Open answer | Open answer- what do authors say about external validity, if anything? | |

## Tool for quasi-experimental designs

| Question | Coding format | Criteria | Decision rule |
| --- | --- | --- | --- |
| General | | | |
| ID | EPPI ID |  |  |
| Time taken to complete assessment | Minutes |  |  |
| Study first author | Open answer |  | |
| Outcome | Open answer |  | |
| Study design: What type of study design is used? | 1= Natural experiment: randomised or as-if randomised 2= Natural experiment: regression discontinuity (RD) 3= CBA (non-randomised assignment with treatment and contemporaneous comparison group, baseline and end line data collection) – individual repeated measurement 4= CBA pseudo panel (repeated measurement for groups but different individuals) 5= Interrupted time series (with or without contemporaneous control group) 6= Panel data, but no baseline (pre-test) 7 = Comparison group with end line data only |  |  |
| Methods used for analysis: Which methods are used to control for selection bias and confounding? | 1 = Statistical matching (PSM, CEM, covariate matching) 2 = Difference in differences (DID) estimation methods 3 = IV-regression (2-stage least squares or bivariate probit) 4 = Heckman selection model 5 = Fixed effects regression 6 = Covariate adjusted estimation 7 = Propensity weighted regression 8 = Comparison of means 9 = Other (please state) |  |  |
| Study population | Open answer | Provide any details in the paper that describe how the study population was selected, covering:  a) How is the population selected? what is the sampling strategy to recruit participants from that population into the study?  b) What are the characteristics of that study participants?  c) Was this a pilot programme aimed at being scaled up?  d) Were there specific factors of success or failure in the implementation? |  |
| Ethical clearance | Open answer | Provide any details of ethical research clearances granted. Report unclear if this information is not available. | |
| Study registration | Open answer | Provide any details of study registration, including registry IDs, etc. | |
| 1: Selection bias - Assessment | | | |
| Mechanism of assignment: was the allocation or identification mechanism able to control for selection bias? | 1= Yes,  2 = Probably Yes,  3 = Probably No,  4 = No,  8 = Unclear | Complete the justification column before answering this column. Use the indications in this row below in the justification column to answer this question.   Here talking about assignment to treatment and control group. |  |
| For regression discontinuity designs | Open answer. Your answer should include all points here (a, b, c), the justification for each point (cite parts from the study) and a page number.  a, Yes as... p.XX b No as...p.XX c Yes as... p.XX | a) Allocation is made based on a pre-determined discontinuity on a continuous variable (regression discontinuity design) and blinded to participants or;     b) if not blinded assignment, individuals reasonably cannot affect the assignment variable in response to knowledge of the participation decision rule;    c) and the sample size immediately at both sides of the cut-off point is sufficiently large to equate groups on average. | Score “Yes” if criteria a), b), c) are all satisfied  Score "Probably Yes" if there are minor differences in between both sides of the cut-off point but authors convincingly argue that the differences are unlikely to affect the outcome, OR individuals are not blinded and there are low risk of them affecting the assignment but the authors do not mention it.  Score “Unclear” if it is unclear whether participants can affect it in response to knowledge of the allocation mechanism.   Score "Probably No" if there are differences between individuals on both sides of the cut-off point, and there are doubts that the differences are due to individuals altering the assignment OR the participants are blinded but there is evidence that the decisions that determined the discontinuity is based on differences between the two groups or differences in time.   Score “No” if the sample size is not sufficient OR there is evidence that participants altered the assignment variable prior to assignment. If the research has serious concerns with the validity of the assignment process or the group equivalence completely fails, we recommend assessing risk of bias of the study using the relevant questions for the appropriate methods of analysis (cross-sectional regressions, difference-in-difference, etc.) rather than the RDDs questions. |
| For assignment based non-randomised programme placement and self-selection (studies using a matching strategy or regression analysis, excluding IV)  RELEVANT FOR ALL QED, except IV and RDD. | Open answer. Your answer should include all points here (a, b, c), the justification for each point (cite parts from the study) and a page number.  a, Yes as... p.XX b No as...p.XX c Yes as... p.XX | a) Participants and non-participants are either matched based on all relevant characteristics explaining participation and outcomes, or;    b) all relevant characteristics are accounted for.**    c) and the data set used contains relevant variable that are measured in a relevant way (i.e. they were not collected for a different purpose initially and therefore are good proxy for some characteristics).    **Accounting for and matching on all relevant characteristics is usually only feasible when the programme allocation rule is known and there are no errors of targeting. It is unlikely that studies not based on randomisation or regression discontinuity can score “YES” on this criterion. There are different ways in which covariates can be taken into account. Differences across groups in observable characteristics can be taken into account as covariates in the framework of a regression analysis or can be assessed by testing equality of means between groups. Differences in unobservable characteristics can be taken into account through the use of instrumental variables (see also question 1.d) or proxy variables in the framework of a regression analysis, or using a fixed effects or difference-in-differences model if the only characteristics which are unobserved are time-invariant | Score “Yes” if a) or b) and c) are satisfied   Score "Probably yes" if a) or b) are addressed for but there is some doubt related to c), OR authors combined statistical matching and difference-in-difference to cope with unobservable differences, OR they only did statistical matching and there was clear rules for selection into the programme (no self-selection).   Score “Unclear” if · it is not clear whether all relevant characteristics (only relevant time varying characteristics in the case of panel data regressions) are controlled.    Score "Probably no" if only a statistical matching was done and there was self-selection into the programme.   Score “No” if relevant characteristics are omitted from the analysis. |
| For identification based on an instrumental variable (IV estimation including Endogenous Treatment effect models) | Open answer. | Score “Yes” if an appropriate instrumental variable is used which is exogenously generated: for example, due to a ‘natural’ experiment or random allocation.    Score "Probably yes" if there is less evidence (no balance table showing differences between the intervention and comparison group).   Score “Unclear” if the exogeneity of the instrument is unclear (both externally as well as why the variable should not enter by itself in the outcome equation).   Score "Probably no" if there is evidence that enrolment in the programme is correlated with a variable that might also have an effect on outcome and on the instrumental variable.   Score “No” if it is clear that the instrument is not exogenous and affect the outcome through other channels than the programme. | |
| 2: Confounding bias - Assessment | | | |
| Group equivalence: was the method of analysis executed adequately to ensure comparability of groups throughout the study and prevent confounding? | 1= Yes,  2 = Probably Yes,  3 = Probably No,  4 = No,  8 = Unclear |  |  |
| For regression discontinuity design | Open answer. Your answer should include all points here (a, b, c), the justification for each point (cite parts from the study) and a page number.  a, Yes as... p.XX b No as...p.XX c Yes as... p.XX | a) The interval for selection of treatment and control group is reasonably small OR authors have weighted the matches on their distance to the cut-off point;  b) and the mean of the covariates of the individuals immediately at both sides of the cut-off point (selected sample of participants and non-participants) are overall not statistically different based on t-test or ANOVA for equality of means; c) Significant differences in covariates of the individuals have been controlled in analysis; and for cluster-assignment, authors control for external cluster-level factors that might confound the impact of the programme. | Score "Yes, if criterion a), b), c) and d) are addressed.   Score "Probably yes" if b) is not addressed but c) is addressed and differences in means are not large.   Score “Unclear” if insufficient details are provided on controls; or if insufficient details are provided on cluster controls.   Score "Probably no" if b) is not addressed (absence of a difference test or balance table) and there are doubt regarding the continuity on both sides of the cut-off point (a).   Score “No” otherwise. |
| For non-randomised trials using difference-in-differences methods of analysis (If BOTH PSM and DID are used, code using this DID column) include ITS here. | Open answer. Your answer should include all points here (a, b, c), the justification for each point (cite parts from the study) and a page number.  a, Yes as... p.XX b No as...p.XX c Yes as... p.XX | a) The authors use a difference-in-differences (or fixed effects) multivariable estimation method and assessment of the parallel trends assumption finds similar trends among treatment and control groups; b) the authors control for a comprehensive set of individual time-varying characteristics, and for cluster-assignment, authors control for external cluster-level factors that might confound the impact of the programme**; c) and the attrition rate is sufficiently low and similar in treatment and control, or the study assesses that drop-outs are random draws from the sample (for example, by examining correlation with determinants of outcomes, in both treatment and comparison groups);     **Knowing allocation rules for the programme – or even whether the non-participants were individuals that refused to participate in the programme, as opposed to individuals that were not given the opportunity to participate in the programme – can help in the assessment of whether the covariates accounted for in the regression capture all the relevant characteristics that explain differences between treatment and comparison | Score "Yes, if a, b, c, d (if relevant) are addressed and baseline imbalances between groups were relatively low OR the method was combined by a statistical matching.   Score "Probably yes" if all possible variables are controlled for and the selection into the programme was done according to clear rules, but baseline imbalances between groups were very large.   Score “Unclear” if insufficient details are provided; or if insufficient details are provided on cluster controls.    Score "Probably no" if some time-varying characteristics are not controlled for and the programme was self-selected by the intervention groups, or there is evidence that trends are not parallel..   Score “No” if any of the criterion is not addressed. |
| For statistical matching studies including propensity scores (PSM) and covariate matching**  **Matching strategies are sometimes complemented with difference-in-difference regression estimation methods. This combination approach is superior since it only uses in the estimation the common support region of the sample size, reducing the likelihood of existence of time-variant unobservable differences across groups affecting outcome of interest and removing biases arising from time-invariant unobservable characteristics. | Open answer. Your answer should include all points here (a, b, c, d), the justification for each point (cite parts from the study) and a page number.  a, Yes as... p.XX b No as...p.XX c Yes as... p.XX d Maybe ... p.XX | a) Matching is either on baseline characteristics or time-invariant characteristics which cannot be affected by participation in the programme; and the variables used to match are relevant (for example, demographic and socio-economic factors) to explain both participation and the outcome (so that there can be no evident differences across groups in variables that might explain outcomes); and, for cluster-assignment, authors control for external cluster-level factors that might confound the impact of the programme b) in addition, for PSM Rosenbaum’s test suggests the results are not sensitive to the existence of hidden bias AND Gamma/threshold is 2 or more;  c) and, with the exception of Kernel matching, the means of the individual covariates are equated for treatment and comparison groups after matching;  d) different matching methods including varying sample sizes yields the same results and authors take into account the use of control observations multiple times against the same treatment in their standard error calculation. | Score "Yes, if a, b, c, and d (if relevant) are addressed.   Score "Probably yes" if the selection into the programme was done according to clear rules, which are used for the matching but there are slight imbalances remaining after matching.    Score “Unclear” if relevant variables are not included in the matching equation, or if matching is based on characteristics collected at end line; or if insufficient details are provided on cluster controls.   Score "Probably no" if the programme was self-selected by the intervention groups or participants OR if the selection into the programme was done according to clear rules but there is no baseline data available to match the participants or groups on.   Score “No” if matching was done based on variables that are likely to be affected by the programme or any other scenario that affect a), b) c) or d). |
| For regression-based studies using cross sectional data (excluding IV) | Open answer. Your answer should include all points here (a, b, c, d), the justification for each point (cite parts from the study) and a page number.  a, Yes as... p.XX b No as...p.XX c Yes as... p.XX d Maybe ... p.XX | a) The study controls for relevant confounders that may be correlated with both participation and explain outcomes (for example, demographic and socio-economic factors at individual and community level) using multivariable methods with appropriate proxies for unobservable covariates, and, for cluster-assignment, authors control particularly for external cluster-level factors that might confound the impact of the programme; b) and a Hausman test with an appropriate instrument suggests there is no evidence of endogeneity**; c) and none of the covariate controls can be affected by participation; d) and either, only those observations in the region of common support for participants and non-participants in terms of covariates are used, or the distributions of covariates are balanced for the entire sample population across groups;    **The Hausman test explores endogeneity in the framework of regression by comparing whether the OLS  and the IV approaches yield significantly different estimations. However, it plays a different role in the  different methods of analysis. While in the OLS regression framework the Hausman test mainly explores  endogeneity and therefore is related with the validity of the method, in IV approaches it explores whether the  author has chosen the best available strategy for addressing causal attribution (since in the absence of  endogeneity OLS yields more precise estimators) and therefore is more related with analysis reporting bias. | Score "Yes, if a, b, c and d are addressed.   Score "Probably yes" if all criterion are addressed but authors did not report the Hausman test (b).   Score “Unclear” if relevant confounders are controlled but appropriate proxy variables or statistical tests are not reported; or if insufficient details are provided on cluster controls.    Score "Probably no" if any of the criterion other than b) is not addressed.   Score “No" if none of the criterion are addressed. |
| For identification based on an instrumental variable (IV estimation including Endogenous Treatment effect models) | Open answer. Your answer should include all points here (a, b, c, d), the justification for each point (cite parts from the study) and a page number.  a, Yes as... p.XX b No as...p.XX c Yes as... p.XX d Maybe ... p.XX | a) The instrumenting equation is significant at the level of F≥10 (or if an F test is not reported, the authors report and assess whether the R-squared (goodness of fit) of the participation equation is sufficient for appropriate identification);   b) the identifying instruments are individually significant (p≤0.01); for Heckman models, the identifiers are reported and significant (p≤0.05);  c) where at least two instruments are used, the authors report on an over-identifying test (p≤0.05 is required to reject the null hypothesis); and none of the covariate controls can be affected by participation and the study convincingly assesses qualitatively why the instrument only affects the outcome via participation. If the instrument is the random assignment of the treatment, the reviewer should also assess the quality and success of the randomisation procedure in part a).  d) and, for cluster-assignment, authors particularly control for external cluster-level factors that might confound the impact of the programme (for example, weather, infrastructure, community fixed effects, and so forth) through multivariable analysis. | Score "Yes, if a, b, c, d (if relevant) are addressed.   Score "Probably yes" if one of the test required for criterion a) or b) is not reported but the other is, and the rest of the criterion are addressed and the instrument is convincing.   Score “UNCLEAR” if relevant confounders are controlled for but appropriate statistical tests are not reported; or if insufficient details are provided on cluster controls   Score "Probably no" if exogeneity of the instrument is not convincing and appropriate tests are not reported.   Score “No” otherwise if any of the tests required for criterion a), b) or c) are reported and not satisfied. |
| 3: Performance bias - Assessment | | | |
| Performance bias: was the process of being observed free from motivation bias? | 1= Yes,  2 = Probably Yes,  3 = Probably No,  4 = No,  8 = Unclear | a) For data collected in the context of a particular intervention trial (randomised or non-randomised assignment), the authors state explicitly that the process of monitoring the intervention and outcome measurement is blinded, or argue convincingly why it is not likely that being monitored could affect the performance of participants in treatment and comparison groups in different ways (such as resulting in Hawthorne or John Henry effects).  b) The study is based on data collected in the context of a survey, and data collection is not associated with a particular intervention (which may increase motivation bias), or data are collected from administrative records or in the context of a retrospective (ex post) evaluation. | Score “Yes” if either criterion a) or b) are satisfied;   Score "Probably yes" if the study is based on survey data collected during a trial and there is no obvious issue with the monitoring processes but authors do not mention potential risks.    Score “Unclear” if it is not clear whether the authors use an appropriate method to prevent Hawthorne and John Henry Effects (e.g. blinding of outcomes and, or enumerators, other methods to ensure consistent monitoring across groups). Hawthorne effects may result where participants know that they are being observed and John Henry Effects may result from participant knowledge of being compared.   Score "Probably no" if there was imbalance in the frequency of monitoring in intervention groups, which might have influenced participants' behaviours.   Score "No" eif neither criterion a) or b) are satisfied; |
| Performance bias - Justification | Open answer. Your answer should include all points here (a, b), the justification for each point (cite parts from the study) and a page number. | Justification for coding decision  (Include a brief summary of justification for rating, mentioning your response to all sub questions, cite relevant pages). | |
| 4: Spill-overs, cross-overs and contamination - Assessment | | | |
| Spill-overs, cross-overs and contamination: was the study adequately protected against spill-overs, cross-overs and contamination? | 1= Yes,  2 = Probably Yes,  3 = Probably No,  4 = No,  8 = Unclear | a) There was no implementation issues that might have led the control participants to receive the treatment (implementer's mistake).  b) The intervention is unlikely to spill-over to comparisons (e.g. participants and non-participants are geographically and/or socially separated from one another and general equilibrium effects are not likely) or the potential effects of spill overs were measured (e.g. variation in the % of unit within a cluster receiving the treatment).  c) There is no risk of contamination by external programmes: the treatment and comparisons are isolated from other interventions which might explain changes in outcomes.   d) There is nothing in the surveys that might have given the control participants an idea of what the other group might receive OR they did but there is no risk that this has changed their behaviours; AND the survey process did not reveal information to the control group that they did not have before (e.g. the study aims to measure increase in take up of a service or product that participants might not know about)  Authors might put something in place in the design of the study that allows to control for that survey effect (e.g. a pure control with no monitoring except baseline end line) | Score “Yes” if criterion a), b), c) and d) are satisfied;    Score "Probably yes" if there is no obvious problem but there is no information reported on potential risks related to spill overs, contamination, or survey effects in the control group OR if there were issues with spill-overs but they were controlled for or measured.    Score “Unclear” if spill-overs, cross-overs, survey effects and/or contamination are not addressed clearly.    Score "Probably no" if any of the criterion a), b), c) or d) are not satisfied but the scale of the issue is not clear.    Score “No” if any of the criterion a), b), c) or d) are not satisfied and happened at a large scale in the study. |
| Spill-overs, cross-overs and contamination - Justification | Open answer | Justification for coding decision  (Include a brief summary of justification for rating, mentioning your response to all sub questions, cite relevant pages). | |
| 5: Outcome measurement bias - Assessment | | | |
| Was the study free from outcome measurement bias? | 1= Yes,  2 = Probably Yes,  3 = Probably No,  4 = No,  8 = Unclear | a) Outcome assessors are blinded or the outcome measures are not likely to be biased by their judgement.   b) For self-reported outcomes: respondents in the intervention group are not more likely to have accurate answers due to recall bias; Length of the recall period for both intervention and control groups is important here. Discuss here how recall data bias may affect the outcome measurement of the intervention or control group (if relevant, criterion NOT satisfied). If it affects both treatment and control groups in a similar way, do not consider this as a risk of bias (criterion satisfied).    c) For self-reported outcomes: respondents do not have incentives to over/under report something related to their performance or actions, OR researchers put in place mechanisms to reduce the risk of reporting bias (researchers not strongly involved in the implementation of the programme and it is clear that their answers to the survey will not affect what they receive in the future) OR authors have measured the risks of bias through falsification tests or measuring the effect on placebo outcomes in cases where there was a risk of reporting bias.  d) Timing issue: the data collection period did not differ between intervention and comparison group, the baseline data is not likely to be affected by the beginning of the intervention or affects a small percentage of the study participants. | Score “Yes” if criterion a), b), c) and d) are satisfied:    Score "Probably yes" if there is a small risk related to any of a), b), c) or d) and there is no more information provided to justify the absence of bias OR if there was a high risk of bias but authors have either controlled it in their design or measured it with a placebo outcomes.    Score “Unclear” if it there is a high risk related to any of a), b), c) or d) and there is no more information provided to justify the absence of bias.     Score "Probably no" if there are high risk related to a), b), c) or d) and it is clear that authors were not able to control for this bias.    Score “No” if there is evidence of bias. |
| Outcome measurement bias - Justification | Open answer. Your answer should include all points here (a, b, c), the justification for each point (cite parts from the study) and a page number.  a, Yes as... p.XX b No as...p.XX c Yes as... p.XX | Justification for coding decision  (Include a brief summary of justification for rating, mentioning your response to all sub questions, cite relevant pages). | |
| 6: Reporting bias - Assessment | | | |
| Selective analysis reporting: was the study free from selective analysis reporting? | 1= Yes,  2 = Probably Yes,  3 = Probably No,  4 = No,  8 = Unclear | a) a pre-analysis plan is published, especially for prospective NRS (non-randomised studies) but it should also be for retrospective studies b) authors use ‘common’ methods of estimation (i.e. credible analysis method to deal with attribution given the data available) ; c) There is no evidence that outcomes were selectively reported (e.g. results for all relevant outcomes in the pre-analysis plan are reported in the results section. If the PAP is not available, then the results for all outcomes discussed in the methods section are reported in the results section.) ; d) Requirements for specific methods of analysis:  - For PSM and covariate matching: (a) Where over 10% of participants fail to be matched, sensitivity analysis is used to re-estimate results using different matching methods (Kernel Matching techniques); (b) For matching with replacement, no single observation in the control group is matched with a large number of observations in the treatment group.  - For IV (including Heckman) models, (a) The authors test and report the results of a Hausman test for exogeneity (p≤0.05 is required to reject the null hypothesis of exogeneity); (b) the coefficient of the selectivity correction term (Rho) is significantly different from zero (P<0.05) (Heckman approach).   - For studies using multivariable regression analysis, authors conduct appropriate specification tests (e.g. testing robustness of results to the inclusion of additional variables, or (very rare) reporting results of multicollinearity test etc). | Score “Yes” if a), b), c) and d) are satisfied OR if a) is not met and it is a retrospective NRS. Score "Probably Yes" if authors combined methods and reported relevant tests (d) only for one method OR if all the criteria are met except for a) and it is a prospective NRS Score "Unclear" if intended outcomes not specified in the paper OR if any of the requirements for d) are not reported.  Score "Probably No" if b) is addressed, but authors did not present results for all outcomes announced in the method section OR did not meet requirement d) although reported.  Score “No” if authors use uncommon or less rigorous estimation methods such as failure to conduct multivariable analysis for outcomes equations OR if some important outcomes are subsequently omitted from the results or the significance and magnitude of important outcomes was not assessed. |
| Analysis reporting bias - Justification | Open answer. Your answer should include all points here (a, b, c, d), the justification for each point (cite parts from the study) and a page number.  a, Yes as... p.XX b No as...p.XX c Yes as... p.XX d Maybe ... p.XX | Justification for coding decision  (Include a brief summary of justification for rating, mentioning your response to all sub questions, cite relevant pages). | |
| 7: Other bias - Assessment | | | |
| Other risks of bias: Was the study free from other sources of bias? | 1= Yes,  4 = No | Score “Yes” if the reported results do not suggest any other sources of bias.  Score “No” if other potential threats to validity are present, and note these here (e.g. coherence of results, survey instruments used are not reported) | |
| Other risks of bias - Justification | Open answer | Justification for coding decision  (Include a brief summary of justification for rating, mentioning your response to all sub questions, cite relevant pages). | |
| 8: External validity | | | |
| External validity | Open answer | Open answer- what do authors say about external validity, if anything? | |

# Appendix 5. Calculating standardised effects

An effect size expresses the magnitude (or strength) and direction of the relationship of interest (Valentine, Aloe, and Lau 2015; Borenstein et al. 2009). We will extract data from each individual study to calculate standardised effect sizes for cross-study comparison wherever possible. For continuous outcomes comparing group means in a treatment and control group, we will calculate the standardised mean difference (SMDs), or Cohen’s d, its variance and standard error using formulae provided in Borenstein et al. (2009). A SMD is a difference in means between the treatment and control groups divided by the pooled standard deviation of the outcome measure. Cohen’s d can be biased in cases where sample sizes are small. Therefore, in all cases we will simply adjust d using Hedges’ method, adjusting Cohen’s d to Hedges’ g using the following formula (Ellis 2010):

$$g\cong d(1-\frac{3}{4(n_{1}+n_{2})-9})$$

We will choose the appropriate formulae for effect size calculations in reference to, and dependent upon, the data provided in included studies. For example, for studies reporting means (X) and pooled standard deviation (SD) for treatment (T) and control or comparison (C) at follow up only:

$$d=\frac{x_{Tp+1}-x_{Cp+1}}{SD}$$

If the study does not report the pooled standard deviation, it is possible to calculate it using the following formula:

$${SD}_{p+1}=\sqrt{\frac{\left( n_{Tp+1}-1 \right){SD}_{Tp+1}^{2}+\left( n_{Cp+1}-1 \right){SD}_{Cp+1}^{2}}{n_{Tp+1}+n_{Cp+1}-2}}$$

Where the intervention is expected to change the standard deviation of the outcome variable, we will use the standard deviation of the control group only.

For studies reporting means $(\underline{X})$ and standard deviations (SD) for treatment and control or comparison groups at baseline (p) and follow up (p+1):

$$d= \frac{{\Delta\underline{X}}_{p+1}-{\Delta\underline{X}}_{p}}{{SD}_{p+1}}$$

For studies reporting mean differences $(\Delta\underline{X})$ between treatment and control and standard deviation (SD) at follow up (p+1):

$$d=\frac{{\Delta\underline{X}}_{p+1}}{{SD}_{p+1}}= \frac{\underline{X}_{Tp+1}-\underline{X}_{Cp+1}}{{SD}_{p+1}}$$

For studies reporting mean differences between treatment and control, standard error (SE) and sample size (n):

$$d=\frac{{\Delta\underline{X}}_{p+1}}{SE\sqrt{n}}$$

As primary studies have become increasingly complex, it has become commonplace for authors to extract partial effect sizes (e.g. a regression coefficient adjusted for covariates) in the context of meta-analysis. For studies reporting regression results, we will follow the approach suggested by Keef and Roberts (2004) using the regression coefficient and the pooled standard deviation of the outcome. Where the pooled standard deviation of the outcome is unavailable, we will use regression coefficients and standard errors or t-statistics to do the following, where sample size information is available in each group:

$$d= t\sqrt{\frac{1}{n_{T}}+\frac{1}{n_{C}}}$$

where n denotes the sample size of treatment group and control. We will use the following where only the total sample size information (N) is available, as suggested in Polanin et al. (2016):

$d=\frac{2t}{\sqrt{N}}$ ${Var}_{d}=\frac{4}{N}+\frac{d^{2}}{4N}$

We will calculate the t-statistic (t) by dividing the coefficient by the standard error. If the authors only report confidence intervals and no standard error, we will calculate the standard error from the confidence intervals. If the study does not report the standard error, but report t, we will extract and use this as reported by the authors. In cases in which significance levels are reported rather than t or SE (b), then t will be imputed as follows:

Prob > 0.1: t = 0.5

0.1 ≥ Prob > 0.05: t = 1.8

0.05 ≥ Prob > 0.01: t = 2.4

0.01 ≥ Prob: t = 2.8

Where outcomes are reported in proportions of individuals, we will calculate the Cox-transformed log odds ratio effect size (Sánchez-Meca, Marín-Martínez, and Chacón-Moscoso 2003):

$$d=LogOddsRatio* \frac{\surd3}{\pi}$$

where OR is the odds ratio calculated from the two-by-two frequency table.

Where outcomes are reported based on proportions of events or days, we will use the standardised proportion difference effect size:

$$d= \frac{p_{T} - p_{C}}{SD\left( p \right)}$$

Where *p_t_* is the proportion in the treatment group and *p_c_* the proportion in the comparison group, and the denominator is given by:

$$SD\left( p \right)= \sqrt{p \left( 1-p \right)}$$

where p is the weighted average of *p_c_* and *p_t_*:

$$p= \frac{n_{T} p_{T} + n_{C} p_{C}}{n_{T}+ n_{C}}$$

An independent reviewer will evaluate a random selection of 10 percent of effect sizes to ensure that the correct formulae were employed in effect size calculations. In all cases after synthesis, we will convert pooled effect sizes to commonly used metrics such as percentage changes and mean differences in outcome metrics typically used (e.g. weight in kg) whenever feasible.

# Appendix 6. Criteria determining selection of effect estimates for data extraction

We will extract effects reported across different interventions, outcomes and subgroups within a study. We will address dependent effect sizes using data processing and selection techniques. We will utilise several criteria to select one effect estimate per outcome per study:

- Where studies report effects from multiple estimators, we will use the author's preferred specification. If no preference is indicated, we will select the estimate based on a model specification with the most controls. If data is available, we will use the specification that appears most robust to falsification tests (e.g. according to sensitivity analysis for propensity score matching or placebo tests for difference-in-difference estimators).
- Where different studies report on the same programme but use different samples (e.g., from different regions), we will include both estimates, treating them as independent samples, provided effect sizes are measured relative to separate control or comparison groups.
- Where studies report evidence according to subgroups of participants, we will record and report data on relevant subgroups separately.
- For studies with outcome measures at different time points, we will synthesise short- and long-term outcomes separately, following De La Rue et al. (2013).
- When a study uses multiple outcome measures for a particular construct, we will use the measure that appears to most accurately capture the construct and without regard to the results reported. In these cases, if authors do not present an effect for the full sample, we may calculate a “synthetic effect size” using the sample-weighted average and applying appropriate formulae to recalculate variances (Borenstein et al. [2009](https://onlinelibrary.wiley.com/doi/10.1002/cl2.1180#cl21180-bib-0013), ch. 24).
- If studies include multiple treatment arms with only one control group and the treatments represent separate treatment constructs, we will calculate the effect size for treatment A versus control and treatment B versus control and include them in separate meta-analyses according to the intervention type. Where multiple treatment arms represent the same treatment construct, we may calculate a “synthetic effect size”.
- Our analysis will prioritise synthesising outcomes using composite or aggregate indicators. If a study does not report a composite measure, we will use the outcome that most closely relates to the intervention type or perform outcome mapping to identify the outcome in each study that appears most frequently across studies.

# Appendix 7. Critical appraisal tool for qualitative studies

The following table provides a provisional critical appraisal tool for qualitative studies, descriptive quantitative studies, mixed-methods studies, and process evaluations. If necessary, we may amend the tool to better inform the appraisal of studies.

| Study type | **Methodological appraisal criteria** | | | | | | | | | | | | | | | **Response** | | | | |
| --- | --- | --- | --- | --- | --- | --- | --- | --- | --- | --- | --- | --- | --- | --- | --- | --- | --- | --- | --- | --- |
|  |  |  |  |  |  |  |  |  |  |  |  |  |  |  |  | Yes | | No | Comment | |
| *Screening questions: assessing ‘fatal flaws’* | Configurative assessment:  Study reports primary data and applied methods  Study states clear research questions and objectives  Study states clear research design, which is appropriate to address the stated research question and objectives (*Purposivity*)  The findings of the study are based on collected data, which justify the knowledge claims (*Accuracy*) | | | | | | | | | | | | | | |  | |  |  | |
|  | ***Screening question based on abstract and/or superficial reading of full text: Further appraisal is not feasible or appropriate when the answer is ‘No’ to any of the above screening questions!*** | | | | | | | | | | | | | | | | | | | |
| Study type | **Methodological appraisal criteria** | | | | | | | | | | | | | | | **Response** | | | | |
|  |  |  |  |  |  |  |  |  |  |  |  |  |  |  |  | Yes | | No | Comment / Confidence judgment | |
| *1. Qualitative and descriptive quantitative, and process evaluations* | **RESEARCH IS DEFENSIBLE IN DESIGN (**providing a research strategy that addresses the question)    Appraisal indicators:    *Is the research design clearly specified and appropriate for the aims and objectives of the research?*    Consider whether | | | | | | | | | | | | | | |  | |  |  | |
|  | *there is a discussion of the rationale for the study design* | | | | | | | | | | | | | | |  | |  |  | |
|  | *the research question is clear, and suited to the inquiry* | | | | | | | | | | | | | | |  | |  |  | |
|  | *there are convincing arguments for different features of the study design* | | | | | | | | | | | | | | |  | |  |  | |
|  | *limitations of the research design and implications for the research evidence are discussed* | | | | | | | | | | | | | | |  | |  |  | |
|  | **Defensible** | **Arguable** | | | **Critical** | | | | | | | | | | **Not defensible** | *Worth to continue:* | | | | |
|  |  | | | | | | | | | | | | | | | | | | | |
|  | **RESEARCH FEATURES AN APPROPRIATE SAMPLE (**following an adequate strategy for selection of participants)    Appraisal indicators:    Consider whether | | | | | | | | | | | | | | |  | |  |  | |
|  | *there is a description of study location and how/why it was chosen* | | | | | | | | | | | | | | |  | |  |  | |
|  | *the researcher has explained how the participants were selected* | | | | | | | | | | | | | | |  | |  |  | |
|  | *the selected participants were appropriate to collect rich and relevant data* | | | | | | | | | | | | | | |  | |  |  | |
|  | *reasons are given why potential participants chose not take part in study* | | | | | | | | | | | | | | |  | |  |  | |
|  | **Appropriate sample** | | **Functional sample** | | | | | **Critical sample** | | | | | | **Flawed sample** | | *Worth to continue:* | | | | |
|  |  | | | | | | | | | | | | | | | | | | | |
|  | **RESEARCH IS RIGOROUS IN CONDUCT**  (Providing a systematic and transparent account of the research process)    Appraisal indicators:    Consider whether | | | | | | | | | | | | | | |  | |  |  | |
|  | *researchers provide a clear account/description of the process by which data was collected (e.g. for interview method, is there an indication of how interviews were conducted?/procedures for collection or recording of data?)* | | | | | | | | | | | | | | |  | |  |  | |
|  | *researchers demonstrate that data collection targeted depth, detail and richness of information (e.g. interview/observation schedule)* | | | | | | | | | | | | | | |  | |  |  | |
|  | *there is evidence of how descriptive analytical categories, classes, labels, etc. have been generated and used* | | | | | | | | | | | | | | |  | |  |  | |
|  | *presentation of data distinguishes clearly between the data, the analytical frame used, and the interpretation* | | | | | | | | | | | | | | |  | |  |  | |
|  | *methods were modified during the study; and if so, has the researcher explained how and why?* | | | | | | | | | | | | | | |  | |  |  | |
|  | **Rigorous conduct** | **Considerate conduct** | | | | | | | **Critical conduct** | | **Flawed conduct** | | | | | *Worth to continue:* | | | | |
|  |  | | | | | | | | | | | | | | | | | | | |
|  | **RESEARCH FINDINGS ARE CREDIBLE IN CLAIM/BASED ON DATA**  (Providing well-founded and plausible arguments based on the evidence generated)    Appraisal indicators:    Consider whether | | | | | | | | | | | | | | |  | |  |  | |
|  | *there is a clear description of the form of the original data* | | | | | | | | | | | | | | |  | |  |  | |
|  | *sufficient amount of data is presented to support interpretations and findings/conclusions* | | | | | | | | | | | | | | |  | |  |  | |
|  | *the researchers explain how the data presented were selected from the original sample to feed into the analysis process (i.e. commentary and cited data relate; there is an analytical context to cited data, not simply repeated description; is there an account of frequency of presented data?)* | | | | | | | | | | | | | | |  | |  |  | |
|  | *there is a clear and transparent link between data, interpretation, and findings/conclusion* | | | | | | | | | | | | | | |  | |  |  | |
|  | *there is evidence (of attempts) to give attention to negative cases/outliers etc.* | | | | | | | | | | | | | | |  | |  |  | |
|  | **Credible claims** | **Arguable claims** | | | | | **Doubtful claims** | | | | **Not credible** | | | | | *If findings not credible, can data still be used?* | | | | |
|  |  | | | | | | | | | | | | | | | | | | | |
|  | **RESEARCH ATTENDS TO CONTEXTS**  (Describing the contexts and particulars of the study)    Appraisal indicators:    Consider whether | | | | | | | | | | | | | | |  | |  |  | |
|  | *there is an adequate description of the contexts of data sources and how they are retained and portrayed?* | | | | | | | | | | | | | | |  | |  |  | |
|  | *participants’ perspectives/observations are placed in personal contexts* | | | | | | | | | | | | | | |  | |  |  | |
|  | *appropriate consideration is given to how findings relate to the contexts (how findings are influenced by or influence the context)* | | | | | | | | | | | | | | |  | |  |  | |
|  | *the study makes any claims (implicit or explicit) that infer generalization (if yes, comment on appropriateness)* | | | | | | | | | | | | | | |  | |  |  | |
|  | **Context central** | **Context considered** | | | | | | **Context mentioned** | | | | | **No context attention** | | |  | | | | |
|  |  | | | | | | | | | | | | | | | | | | | |
|  | **RESEARCH IS REFLECTIVE**  (Assessing what factors might have shaped the form and output of research)    Appraisal indicators:    Consider whether | | | | | | | | | | | | | | |  | |  |  | |
|  | *appropriate consideration is given to how findings relate to researchers’ influence/own role during analysis and selection of data for presentation* | | | | | | | | | | | | | | |  | |  |  | |
|  | *researchers have attempted to validate the credibility of findings (e.g. triangulation, respondent validation, more than one analyst)* | | | | | | | | | | | | | | |  | |  |  | |
|  | *researchers explain their reaction to critical events that occurred during the study* | | | | | | | | | | | | | | |  | |  |  | |
|  | *researchers discuss ideological perspectives/values/philosophies and their impact on the methodological or other substantive content of the research (implicit/explicit)* | | | | | | | | | | | | | | |  | |  |  | |
|  | **Reflection** | **Consideration** | | | | **Acknowledgement** | | | | | | **Unreflective research** | | | | | *NB: Can override previous exclusion!* | | | |
| OVERALL CRITICAL APPRAISAL DECISION  Decision rule:  - a single critical appraisal judgement in any of the 6 appraisal domains leads to a critical overall judgement.  - 2 or more high critical appraisal judgements in any of the 6 appraisal domains lead to an overall high risk of bias / low quality rating.  - 2 or more moderate critical appraisal judgements in any of the 6 appraisal domains lead to an overall moderate risk of bias / moderate quality rating.  - which means that for a study to be rated of low risk of bias / high quality at least 5 appraisal domains need be rated as of low critical appraisal. | | | | | | | | | | | | | | | | | | | | |
| HIGH QUALITY  EMPIRICAL RESEARCH    (Study generates new evidence relevant to the review question and complies with all methodological criteria to ensure reliability and empirical grounding of the evidence). | **MODERATE QUALITY**  **EMPIRICAL RESEARCH**    (Study generates new evidence relevant to the review question and complies with reasonable methodological criteria to ensure reliability and empirical grounding of the evidence). | | | | | | | | | **LOW QUALITY**  **EMPIRICAL RESEARCH**    (Study generates new evidence relevant to the review question and complies with minimum methodological criteria to ensure reliability and empirical grounding of the evidence). | | | | | | | | **CRITICAL QUALITY**  **EMPIRICAL RESEARCH**    (The evidence generated by the study does not comply with minimum methodological criteria to ensure reliability and empirical grounding of the evidence). | | |
|  | | | | | | | | | | | | | | | | | | | | |
| Study type | | | | **Methodological appraisal criteria** | | | | | | | | | | | | | | **Response** | | |
|  |  |  |  |  |  |  |  |  |  |  |  |  |  |  |  |  |  | Yes | No | Comment /confidence judgment |
| *2. Mixed-methods*    *Sequential explanatory design*  *The quantitative component is followed by the qualitative. The purpose is to explain quantitative results using qualitative findings. E.g., the quantitative results guide the selection of qualitative data sources and data collection, and the qualitative findings contribute to the interpretation of quantitative results.*  *Sequential exploratory design the qualitative component is followed by the quantitative. The purpose is to explore, develop and test an instrument (or taxonomy), or a conceptual framework (or theoretical model). E.g., the qualitative findings inform the quantitative data collection, and the quantitative results allow a generalization of the qualitative findings.*  *Triangulation designs the qualitative and quantitative components are concomitant. The purpose is to examine the same phenomenon by interpreting qualitative and quantitative results (bringing data analysis together at the interpretation stage), or by integrating qualitative and quantitative datasets (e.g., data on same cases), or by transforming data (e.g., quantization of qualitative data).*  *Embedded/convergent design The qualitative and quantitative components are concomitant. The purpose is to support a qualitative study with a quantitative sub-study (measures), or to better understand a specific issue of a quantitative study using a qualitative sub-study, e.g., the efficacy or the implementation of an intervention based on the views of participants.* | | | | **RESEARCH INTEGRATION/SYNTHESIS OF METHODS**  (Assessing the value-added of the mixed-methods approach)    Applied mixed-methods design:    Sequential explanatory design  Sequential explorative design  Triangulation design  Embedded design      Appraisal indicators:    Consider whether | | | | | | | | | | | | | |  |  |  |
|  |  |  |  | *the rationale for integrating qualitative and quantitative methods to answer the research question is explained*  *[DEFENSIBLE]* | | | | | | | | | | | | | |  |  |  |
|  |  |  |  | *the mixed-methods research design is relevant to address the qualitative and quantitative research questions, or the qualitative and quantitative aspects of the mixed methods research question*  *[DEFENSIBLE]* | | | | | | | | | | | | | |  |  |  |
|  |  |  |  | *there is evidence that data gathered by both research methods was brought together to inform new findings to answer the mixed-methods research question (e.g. form a complete picture, synthesize findings, configuration)*  *[CREDIBLE]* | | | | | | | | | | | | | |  |  |  |
|  |  |  |  | *the approach to data integration is transparent and rigorous in considering all findings from both the qualitative and quantitative module (danger of cherry-picking)*  *[RIGOROUS]* | | | | | | | | | | | | | |  |  |  |
|  |  |  |  | *appropriate consideration is given to the limitations associated with this integration, e.g., the divergence of qualitative and quantitative data (or results)?*  *[REFLEXIVE]* | | | | | | | | | | | | | |  |  |  |
| For mixed-methods research studies, each component undergoes its individual critical appraisal first. Since qualitative studies are either included or excluded, no combined risk of bias assessment is facilitated, and the assigned risk of bias from the quantitative component similarly holds for the mixed-methods research.    The above appraisal indicators only refer to the applied mixed-methods design. If this design is not found to comply with each of the four mixed-methods appraisal criteria below, then the quantitative/qualitative components will individually be included in the review: | | | | | | | | | | | | | | | | | | | | |
| Mixed-methods critical appraisal:  Research is defensible in design  Research is rigorous in conduct  Research is credible in claim  Research is reflective | | | | Qualitative critical appraisal:  Include / Exclude | | | | | | | | | | | | Quantitative critical appraisal:  Low risk of bias  Risk of bias  High risk of bias  Critical risk of bias | | | | |
| Combined appraisal:  Include / Exclude mixed-methods findings judged with ____________________________ risk of bias | | | | | | | | | | | | | | | | | | | | |
|  |  |  |  |  |  |  |  |  |  |  |  |  |  |  |  |  |  |  |  |  |

# Appendix 8. Reliability classification tool for cost evidence

We will appraise how reliable is the cost data identified for each of the programmes included in the review based on the following categories (drawn from Acharya et al. 2024).

**Rank 1 - Gold Standard**

- Reports detailed ingredients costs for major programme components
- Utilises activity-based costing (ABC) methodology where costs are traced to specific activities
- Ingredients are costed for each activity with market prices (if there is no market failure, although many goods may not have multiple producers) or shadow prices when appropriate
- Clear overhead cost allocation using rational allocation bases (e.g., square footage for facility costs, direct labour hours for supervision), see guidelines in the 3iE manual
- Proper implementation of discounting and depreciation for capital costs:
  - Uses appropriate depreciation methods (straight-line, declining balance, or exponential decay) based on asset type
  - Applies correct useful life estimates for different capital assets
  - Includes opportunity costs of capital
  - Accounts for salvage value when applicable
- Economic costs used for all components, including:
  - Volunteer time valued at market rates
  - Donated facilities at market rental rates
  - In-kind contributions are properly valued
  - Opportunity costs included

**Rank 2 - Reliable**

- Components cost using accounting costs for ingredients
- Capital costs are divided using informal methods such as:
  - Simple straight-line depreciation without consideration of opportunity costs
  - Basic allocation of shared facility costs
  - Standard overhead rates without detailed analysis
  - Accounting costs of capital or simple division of purchase price across time
- Time allocation based on staff estimates rather than time studies
- Basic adjustment for inflation but may lack sophisticated price indexing
- Includes major direct costs but may have simplified treatment of indirect costs

**Rank 3 - Medium Reliable**

- Recognition of various programme components
- Costs estimated from other studies that may have used ABC:
  - Uses comparable programme costs with basic adjustments
  - Extrapolates from similar interventions
  - Applies cost data from different contexts with some adaptation, basically from ABC approaches
- Uses actual previous budget expenditures rather than allocations
- May include:
  - Basic salary and wage costs
  - Simple equipment costs
  - Operating expenses without a detailed breakdown
- Limited consideration of overhead allocation

**Rank 4 - Marginal Reliable**

- Reports on actual spent budget rather than allocations, aggregated
- Distinguishes cost of administrative implementation work
- Limited detailed cost analysis
- Typical characteristics:
  - Aggregate cost categories without ingredient detail
  - Missing or unclear overhead allocations
  - Incomplete capital cost treatment
  - No consideration of volunteer or in-kind contributions
  - Lack of systematic cost allocation methods

**Rank 5 - Nearly Unacceptable**

- Only reports basic budget figures
- Lacks component breakdown
- No detailed cost analysis
- Common problems:
  - Only total programme costs were reported
  - No distinction between capital and recurring costs
  - Missing significant cost components
  - No documentation of cost allocation methods
  - Budget figures without actual expenditure verification
  - Absence of any systematic costing approach

# Appendix References

Acharya, A., Hammaker, J., & Glandon, D. (2024). *Measuring cost-effectiveness in impact evaluation*. International Initiative for Impact Evaluation (3ie). https://doi.org/10.23846/WP0061

Borenstein, M., Hedges, L. V., & Rothstein, H. R. (2009). *Introduction to Meta-Analysis*. John Wiley & Sons, Ltd.

De La Rue, L., Polanin, J. R., Espelage, D. L., & Pigott, T. D. (2013). PROTOCOL: School‐based Interventions to Reduce Dating and Sexual Violence: A Systematic Review. *Campbell Systematic Reviews*, *9*(1), 1–43. https://doi.org/10.1002/CL2.106

Ellis, P. D. (2010). *The Essential Guide to Effect Sizes: Statistical Power, Meta-Analysis, and the Interpretation of Research Results*. Cambridge University Press. https://doi.org/10.1017/CBO9780511761676

Keef, S. P., & Roberts, L. A. (2004). The meta‐analysis of partial effect sizes. *British Journal of Mathematical and Statistical Psychology*, *57*(1), 97–129. https://doi.org/10.1348/000711004849303

Polanin, J. R., Tanner-Smith, E. E., & Hennessy, E. A. (2016). Estimating the Difference Between Published and Unpublished Effect Sizes: A Meta-Review. *Review of Educational Research*, *86*(1), 207–236. https://doi.org/10.3102/0034654315582067

Sánchez-Meca, J., Marín-Martínez, F., & Chacón-Moscoso, S. (2003). Effect-Size Indices for Dichotomized Outcomes in Meta-Analysis. *Psychological Methods*, *8*(4), 448–467. https://doi.org/10.1037/1082-989X.8.4.448

Valentine, J. C., Aloe, A. M., & Lau, T. S. (2015). Life After NHST: How to Describe Your Data Without “p-ing” Everywhere. *Basic and Applied Social Psychology*, *37*(5), 260–273. https://doi.org/10.1080/01973533.2015.1060240

Wooldridge, J. M. (2021). *Two-Way Fixed Effects, the Two-Way Mundlak Regression, and Difference-in-Differences Estimators*. Social Science Research Network. https://www.ssrn.com/abstract=3906345
